# Detergents alter the stability and lipid binding properties of the CD1d immunoreceptor

**DOI:** 10.1002/pro.70417

**Published:** 2025-12-22

**Authors:** Uri Z. Miles, M. G. Finn, Andrew C. McShan

**Affiliations:** ^1^ School of Chemistry and Biochemistry Georgia Institute of Technology Atlanta Georgia USA; ^2^ School of Biological Sciences Georgia Institute of Technology Atlanta Georgia USA

**Keywords:** biophysics, detergents, immunoreceptor, lipid protein interactions, lipids, proteins, structure modeling

## Abstract

CD1d, a non‐classical immunoreceptor, plays a central role in lipid antigen presentation to a variety of T cell types. Despite the widespread use of detergents to measure and manipulate lipid/CD1d interactions in vitro, in situ, and in vivo, the molecular basis by which detergents influence the stability of CD1d and its lipid binding properties remains poorly understood. We evaluated the ability of human CD1d to associate with a panel of 13 structurally and chemically diverse detergents spanning non‐ionic, anionic, cationic, and zwitterionic classes. Through conventional intrinsic tryptophan fluorescence binding assays, complemented by the application of microscale thermophoresis and nano differential scanning fluorimetry, we quantified detergent/CD1d binding affinities and evaluated their impact on CD1d thermal stability. In silico modeling with the machine learning‐based tool Chai‐1 provided plausible detergent binding modes, which mirror docking orientations observed for native lipid ligands within the antigen binding groove. High‐affinity detergents were shown to exhibit the capacity to block lipid binding in a lipid‐dependent manner, implicating features that modulate access to the CD1d groove. These findings provide mechanistic insights into detergent‐mediated modulation of CD1d structure, stability, and function, and offer a quantitative framework for optimizing lipid‐loading protocols, detergent extraction approaches, and lipid antigen‐based immunological assays.

## 1 INTRODUCTION

The CD1 immunoreceptor is a non‐classical major histocompatibility complex molecule expressed on the surfaces of hematopoietic cells (i.e., thymocytes and professional antigen‐presenting cells) and non‐hematopoietic cells (i.e., hepatocytes and intestinal epithelial cells), where it presents lipid antigens to *αβ*, *γδ*, and natural killer T cells (Canchis et al., 1993; Evans & Barral, 2024; Luoma et al., 2014; Macho‐Fernandez & Brigl, 2015). As a result of lipid antigen presentation by CD1, T cells possessing lipid‐restricted T cell receptors (TCRs) are triggered, initiating immune responses against pathogenic infections, autoimmunity, and cancer (Barral & Brenner, 2007; Rossjohn et al., 2015). The CD1 family of proteins is composed of five structurally similar isoforms: CD1a, CD1b, CD1c, CD1d, and CD1e (Adams, 2014). Each CD1 isoform presents lipid antigens at the cell surface, with the exception of CD1e, which instead serves as an intracellular lipid transfer protein (Facciotti et al., 2011; Garcia‐Alles et al., 2011). The properly folded CD1 complex is assembled in the endoplasmic reticulum, resulting in a heterotrimeric complex composed of a lipid antigen, the CD1 heavy chain, and non‐covalently associated invariant *β*2‐microglobulin light chain (*β*2m), which, when assembled, traverses through the secretory pathway for cell surface presentation (Moody & Cotton, 2017; Park et al., 2004; Zeng et al., 1997).

The CD1 lipid antigen repertoire has been shown to be largely comprised of long‐chain lipidic antigens characterized by polar, hydrophilic head groups and non‐polar, hydrophobic tail groups (Cheng et al., 2024; Cox et al., 2009; Haig et al., 2011; Huang et al., 2023; Koch et al., 2005; Szoke‐Kovacs et al., 2024; Yuan et al., 2009). All CD1 isoforms have A′ and F′ antigen binding pockets, with each isoform differing in the size and chemistry of each pocket (Adams, 2014; Kaczmarek et al., 2017; Ly & Moody, 2014; Moody et al., 2005; Rathakrishnan & McShan, 2023). The A′ pocket is usually deeper and can accommodate longer lipid tails, while the F′ pocket is typically shallower, accommodating shorter lipid tails and aromatic rings (Adams, 2014; Kaczmarek et al., 2017; Ly & Moody, 2014; Moody et al., 2005; Rathakrishnan & McShan, 2023). Lipid antigen binding often involves aliphatic, hydrophobic hydrocarbon chains of lipid antigens forming Van der Waals interactions with non‐polar, hydrophobic CD1 amino acid residues in the antigen binding groove (Gadola et al., 2002; Koch et al., 2005; Scharf et al., 2010). The polar, hydrophilic head groups of lipid antigens which include carbohydrate, phosphate, or sulfate groups, bind near the entrance portal to the binding groove in between CD1's *α*1/*α*2 helices, which are characterized by an increased frequency of polar, hydrophilic amino acid residues that form favorable hydrogen bonds and salt bridges with the polar portions of lipid antigens (Gadola et al., 2002; Koch et al., 2005; Rathakrishnan & McShan, 2023; Scharf et al., 2010). As a result, the TCR recognizes specific lipid head groups, usually solvent‐exposed polar, hydrophilic atoms that protrude out of the CD1 binding groove (Adams, 2014; Kaczmarek et al., 2017; Ly & Moody, 2014; Moody et al., 2005). Given the vast diversity of CD1‐associated lipids, differences in lipid orientation within the CD1 groove, and the wide array of T cell receptor recognition modes, there is a need to characterize the underlying biophysical parameters and lipid structural elements dictating lipid/CD1 antigen presentation.

Detergents are frequently used for in vitro, in situ, and in vivo studies of CD1. Detergent and surfactant molecules solubilize lipid antigens, either chemically synthesized or extracted from cells, toward (i) “loading” of CD1 molecules with target lipid antigens for tetramer‐based T cell staining assays, (ii) resuspension of lipids for assays of T cell activation and proliferation of peripheral blood mononuclear cells, and (iii) in vitro lipid affinity measurements with recombinant CD1 (Benlagha et al., 2000; Cantu et al., 2003; Huang et al., 2023; Matsuda et al., 2000; Paletta et al., 2015; Pereira et al., 2019; Sidobre & Kronenberg, 2002; Wang et al., 2010). Additionally, detergents are used for the extraction of membrane‐embedded CD1 for mass spectrometry‐based lipid antigen discovery (Benlagha et al., 2000; Cantu et al., 2003; Huang et al., 2023; Matsuda et al., 2000; Paletta et al., 2015; Pereira et al., 2019; Sidobre & Kronenberg, 2002; Wang et al., 2010). As a result of their lipid‐like properties, detergents have been shown to associate with CD1 proteins to displace endogenous or exogenous lipid antigens within the groove. Examples of commonly used detergents to study CD1 include Tween 20, Tyloxapol, CHAPS, CTAB, and Triton X‐100 (Gadola et al., 2002; Im et al., 2009; Le Nours et al., 2016; Mansour et al., 2016; Uldrich et al., 2013). The ability of each detergent to associate with CD1 depends on many factors, including the detergent structure/chemistry, the CD1 isoform, and the CD1 ortholog. For example, it was previously shown that Triton X‐100 and Tween 20, but not CHAPS, could efficiently strip lipid antigens away from CD1c (Mansour et al., 2016). In a separate study, it was reported that Tyloxapol was more efficient than Tween 20 and Triton X‐100 at loading *α*‐galactosylceramide (*α*‐GalCer) analogues onto CD1d, and species‐dependent observations were also made (Paletta et al., 2015). Despite the widespread use of detergents to study CD1, there have been limited attempts to probe the biophysical basis of detergent influence on the lipid binding properties of CD1. A deeper understanding of these interactions would clarify the molecular basis of CD1 lipid and detergent specificity as well as guide the design of optimized lipid‐loading strategies, detergent extraction protocols, and lipid antigen‐based immunological assays.

**FIGURE 1:**
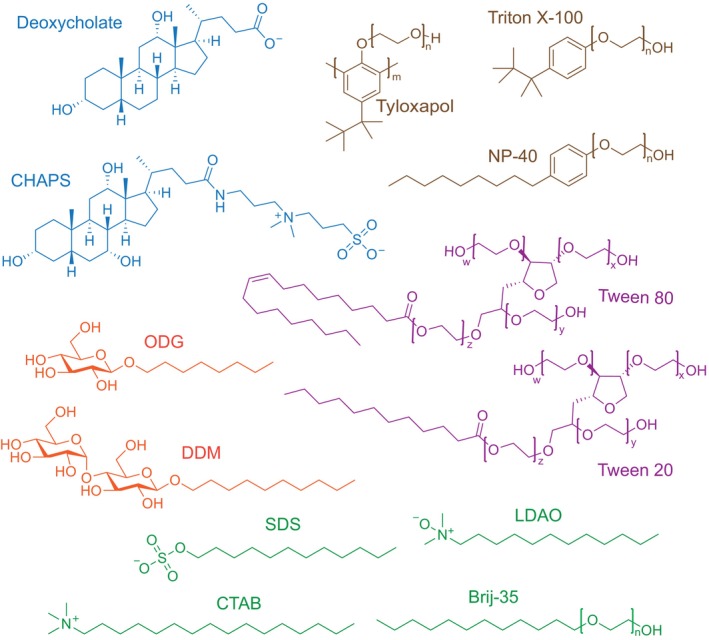
Chemical structures of common detergents used to solubilize lipids. Each molecule is colored by class: Sterol‐like detergents in blue, aromatic detergents in brown, glycosylated detergents in red, PEGylated sorbitols in purple, and aliphatic detergents in green. The “n”, “m”, “x”, “y”, and “z” subscripts represent the average number of repeating groups.

In this work, we explored the ability of human CD1d, one of the most studied CD1 molecules (Cantu et al., 2003; Hong et al., 1999; Koch et al., 2005), to associate with a panel of 13 detergents commonly used to solubilize lipids. The chosen detergents, which sample a diverse range of chemical structures, molecular weights, and critical micelle concentration (CMC) values, were classified as non‐ionic (Tween 20, Tween 80, ODG, Triton X‐100, DDM, Tyloxapol, NP‐40, Brij‐35), anionic (Deoxycholate, SDS), cationic (CTAB), and zwitterionic (LDAO, CHAPS) (Figure 1 and Table 1). A suite of biophysical assays, including intrinsic tryptophan fluorescence (ITF), along with newly developed applications of microscale thermophoresis (MST) and nano differential scanning fluorimetry (nanoDSF) for the CD1 system, were used to quantify detergent/CD1d dissociation constants and assess detergent‐induced destabilization of recombinant human CD1d. In silico modeling with the machine‐learning‐based tool Chai‐1 provided molecular insights into detergent/CD1d binding modes, which were found to be consistent with lipid docking into the antigen‐binding groove. Finally, high‐affinity detergents were shown to block lipid binding to CD1d in a lipid‐dependent manner. Together, these results provide new mechanistic insights into detergent/CD1d interactions, elucidating how detergent class influences antigen displacement (or lack of) and CD1d stability. The results also inform strategies for improving lipid‐loading protocols and detergent‐based extraction methods, with direct implications for the design of lipid antigen formulations, development of CD1d‐targeted immunotherapies, and delivery of lipid‐based vaccine strategies.

**TABLE 1:** Biophysical parameters for different detergents with hCD1d as determined by ITF, MST, and NanoDSF.

Detergent	Mw (g/mol)	Class	CMC μM (%)	*K*_D_ (ITF)	*K*_D_ (MST)	Δ*T*_m_ (NanoDSF)
Tween 20^a^	1226	P/N	59 (0.0072%)	4.9 ± 0.7 μM	1.8 ± 0.5 μM	−8.3 ± 0.2°C
Tween 80^a^	1310	P/N	12 (0.0016%)	4.6 ± 0.9 μM	3.2 ± 0.1 μM	−9.7 ± 0.4°C
n‐octyl‐*β*‐D‐glucopyranoside (ODG)^a^	292.37	G/N	18,000 (0.53%)	>10,000 μM	6579.7 ± 186.0 μM	−0.8 ± 0.4°C
Triton X‐100^a^	625	Ar/N	230 (0.015%)	N/D	45.8 ± 7.9 μM	N/D
n‐dodecyl‐*β*‐D‐maltoside (DDM)^a^	510.62	G/N	170 (0.0087%)	51.4 ± 12.5 μM	35.3 ± 20.5 μM	−12.4 ± 0.8°C
Tyloxapol^c^	Variable	Ar/N	18	N/D	0.9 ± 0.2 μM	N/D
NP‐40^b^	617	Ar/N	290 (0.0179%)	N/D	15.1 ± 1.5 μM	N/D
Brij‐35^a^	1198	Al/N	91 (0.011%)	8.4 ± 4.9 μM	1.7 ± 0.5 μM	−11.4 ± 0.6°C
Hexadecyltrimethylammonium bromide (CTAB)^c^	364.45	Al/C	920 (0.033%)	41.5 ± 6.5 μM	10.9 ± 1.2 μM	−14.2 ± 0.8°C
Sodium deoxycholate^a^	392.58	S/A	6000 (0.24%)	>10,000 μM	>10,000 μM	−10.0 ± 0.3°C
Sodium dodecyl sulfate (SDS)^a^	288.37	Al/A	1990 (0.057%)	35.5 ± 22.6 μM	>10,000 μM	−13.1 ± 0.7°C
Lauryldimethylamine‐N‐oxide (LDAO)^a^	229.41	Al/Z	1000 (0.023%)	>10,000 μM	156.8 ± 58.6 μM	−13.4 ± 0.1°C
3‐((3‐cholamidopropyl) dimethylammonio)‐1‐propanesulfonate (CHAPS)^a^	614.88	S/Z	8000 (0.49%)	<400 μM	< 200 μM	−1.3 ± 0.4°C

*Note*: The approximate CMC is given in both μM and % (weight/volume) values. Detergent classes: N = Non‐ionic, A = Anionic, Z = Zwitterionic, C = Cationic, S = sterol‐like detergents, Ar = aromatic detergents, G = glycosylated detergent, P = PEGylated sorbitols in purple, Al = aliphatic detergents. Dissociation constant (K_D_); N/D = K_D_ not determined due to overlap of fluorescence properties with Trp. Δ*T*_m_ is defined as the *T*_m_ difference between hCD1d in the absence and presence of 1000 μM detergent.

^a^ CMC data from Anatrace catalogue.

^b^ CMC data from Fisher catalogue.

^c^ CMC from the Sigma Aldrich catalogue.

## 2 RESULTS

### 2.1 ITF reveals differences in detergent binding to hCD1d

**FIGURE 2:**
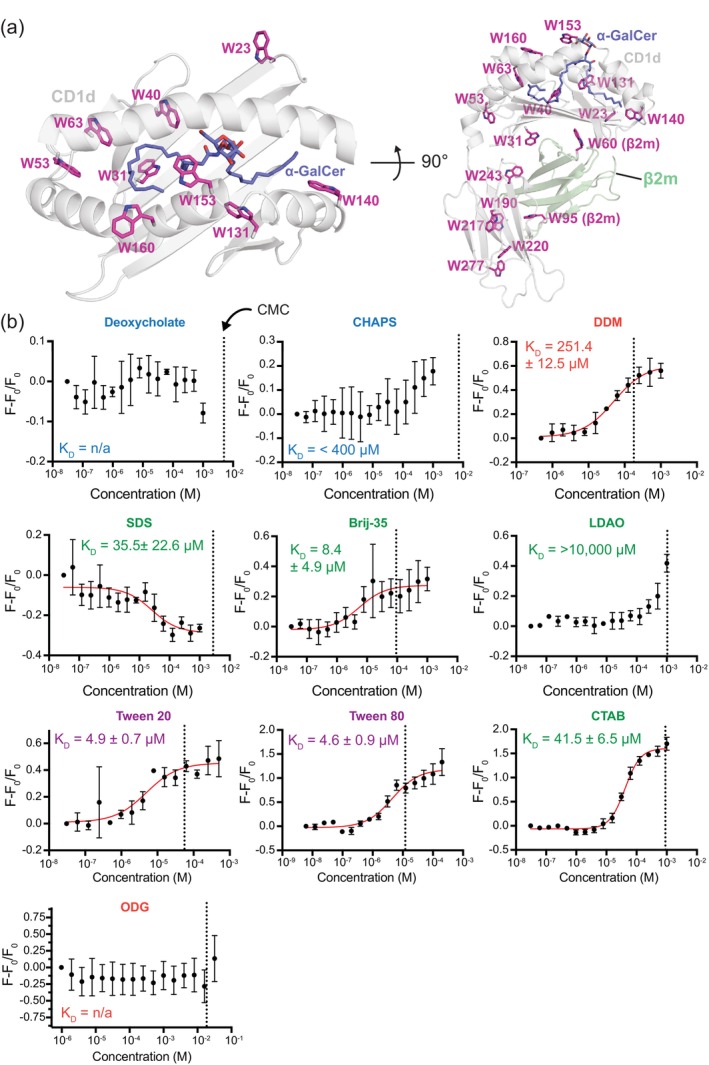
Measurements of detergent binding to hCD1d with ITF. (a) Left: Top view of the *α*‐GalCer/hCD1d antigen binding groove. Right: Side view of the *α*‐GalCer/hCD1d/*β*2m complex. In both views, the locations of tryptophan (Trp) residues (pink sticks) are shown. CD1d and *β*2m are shown as cartoons colored gray and green, respectively. The *α*‐GalCer lipid is colored as blue sticks. The x‐ray structure was taken from PDB ID 1ZT4 (Koch et al., 2005). (b) ITF data showing the change in Trp fluorescence (*F* − *F*_0_/*F*_0_ where *F*_0_ is initial fluorescence in the absence of detergent) as a function of increasing concentrations of detergents in the presence of 100 nM hCD1d (black circles) at 25°C. Fits of the binding isotherms are as shown with a red line. Each data point is mean ± standard deviation for three replicates. The error in dissociation constant (*K*_D_) is derived from the mean ± standard deviation of the individual fits of the three replicates. Detergent names are colored by class, as in Figure 1. Dotted vertical lines represent the CMC values for each detergent (Table 1).

We assessed the binding of each detergent to recombinant human CD1d ectodomain/*β*2m complex (herein called hCD1d), which was expressed as a soluble single‐chain construct in mammalian culture as previously described (Figure S1a,b) (Im et al., 2004). Circular dichroism (CD) spectroscopy and nanoDSF revealed recombinant hCD1d to be properly folded with a melting temperature (*T*_m_) of ~66°C (Figure S1c,d). As a first approach, the binding of detergents to hCD1d was performed with a conventional ITF assay (Cantu et al., 2003; López‐Sagaseta et al., 2012; Scharf et al., 2010). ITF, which does not require modification of either binding partner, is a particularly attractive assay in this case because the hCD1d/β2m complex contains 16 Trp residues; eight are located within the antigen binding groove (Figure 2a). Since intrinsic tryptophan (Trp) fluorescence is highly sensitive to its local chemical environment (Yammine et al., 2019), the binding of ligands into the CD1 binding groove has the potential to either quench Trp fluorescence (due to hydrophobic packing effects) or increase Trp fluorescence (due to ligand‐induced conformational changes toward a more polar environment) (Vivian & Callis, 2001). Second, ITF does not require modification of either CD1 or the lipid/detergent to measure dissociation constants (*K*_D_). Third, the hCD1d/*β*2m complex contains 16 Trp residues; eight are located within the antigen binding groove (Figure 2a). However, while ITF has been used to approximate the apparent affinity of lipids for the hCD1 groove (Cantu et al., 2003; López‐Sagaseta et al., 2012; Scharf et al., 2010), any lipid or detergent that destabilizes or denatures CD1 would also cause changes in Trp fluorescence as the protein unfolds. In such cases, ITF does not necessarily correspond to a true binding event that maintains the properly folded structure (Ghisaidoobe & Chung, 2014). This is one reason why we compare ITF with MST and nanoDSF, as described below.

For ITF assays, the concentration of hCD1d was kept constant, and intrinsic Trp fluorescence (excitation at 285 nm, emission at 335 nm) was measured as a function of increasing detergent concentration (Fossum et al., 2023) (Figure S2). The change in relative fluorescence intensity (*F* − *F*_0_/*F*_0_) provides an indication of binding. Many detergents showed high to moderate apparent affinity to hCD1d with fitted *K*_D_ values between 4 and 300 μM (Figures 2b, S2, and Table 1). The detergents with the highest affinity to hCD1d, as determined by ITF, were Tween 20 (4.9 ± 0.7 μM), Tween 80 (4.6 ± 0.9 μM), and Brij‐35 (8.4 ± 4.9 μM). Some detergents, such as LDAO and CHAPS, exhibited observable binding to hCD1d, but *K*_D_ values could not be determined within the tested concentration range (Figures 2b, S2, and Table 1). This was due to limited detergent solubility and decreased hCD1d stability in the presence of a large excess of detergent (see below). No significant binding was observed for deoxycholate or ODG by ITF. In all but one case, the Trp fluorescence intensity of hCD1d increased, likely corresponding to conformational changes of the Trp residues in the antigen‐binding groove (Cantu et al., 2003; López‐Sagaseta et al., 2012; Scharf et al., 2010). SDS was the exception, for which Trp fluorescence of hCD1d decreased with added detergent. This could indicate a unique conformational change or destabilization of the protein. Three detergents [Tyloxapol (Ding et al., 2017), Triton X‐100 (Zhao & Wei, 2006), and NP‐40] could not be evaluated by ITF due to overlap with the excitation and/or emission spectral region of Trp. Together, the ITF data reveal stark differences in apparent detergent binding to hCD1d with Tween 20, Tween 80, and Brij‐35 as the strongest binders, and deoxycholate and ODG as the weakest binders.

### 2.2 MST reveals differences in detergent binding to hCD1d

The experimental limitations of ITF, such as low instrument sensitivity, high sample consumption, signal overlap with some ligands, and low throughput (Fossum et al., 2023), made it necessary to employ alternative, complementary assays to quantify detergent binding to hCD1d. MST has been used to probe detergent/protein and lipid/protein interactions (Bharambe & Ramachandran, 2020; Sparks et al., 2022), but has not yet been applied to CD1 proteins. MST measures the diffusion of fluorescent molecules in response to microscopic temperature gradients (Seidel et al., 2013). When a protein forms a complex with a ligand, changes in hydrodynamic properties, charge, or solvation energy alter its thermophoretic behavior, allowing detection of binding events. To perform MST experiments, Alexa Fluor 647 (AF647) dye was covalently attached to the primary amines of hCD1d via standard N‐hydroxysuccinimide coupling chemistry (Anderson et al., 1964). The concentration of AF647‐hCD1d was kept constant, and the change in MST signal was measured as a function of increasing detergent concentration (Figure S3). Like ITF, any lipid or detergent that destabilizes CD1 can also alter the MST signal since unfolding changes the protein's Stokes radius and its thermophoretic behavior. Therefore, shifts in the MST signal do not necessarily indicate a bona fide binding event that preserves the native structure. This is why we compare MST results with ITF and nanoDSF, as described below.

**FIGURE 3:**
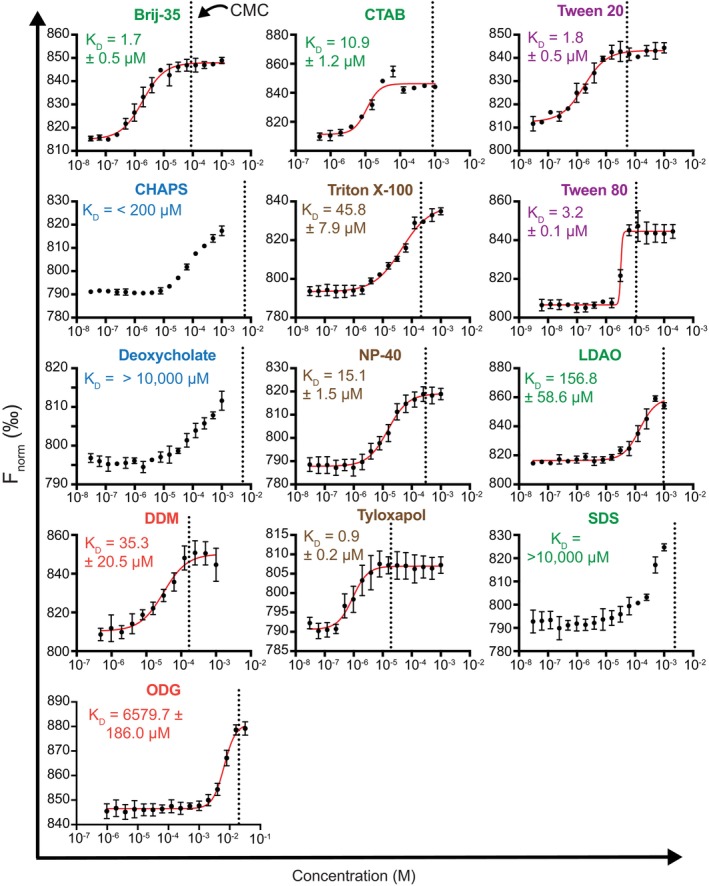
Measurements of detergent binding to hCD1d with MST. MST data showing the change in normalized fluorescence signal (*F*_norm_, black circles) as a function of increasing concentrations of detergents with 100 nM AF647‐labeled hCD1d at 25°C. Fits of the binding isotherms are shown with a red line. Each data is mean ± standard deviation for three replicates. The error in *K*_D_ is derived from the mean ± standard deviation of the individual fits of the three replicates. Detergent names are colored by class, as in Figure 1. Dotted vertical lines represent the CMC values for each detergent (Table 1).

In agreement with ITF, MST showed that many detergents have strong to moderate apparent affinity for hCD1d with determined *K*_D_ values between ~1 and 7000 μM (Figure 3 and Table 1). The detergents with the highest affinity to hCD1d, as determined by MST, were Tyloxapol (0.9 ± 0.2 μM), Brij‐35 (1.7 ± 0.5 μM), Tween 20 (1.8 ± 0.5 μM), and Tween 80 (3.2 ± 0.1 μM). NP‐40 and Triton X‐100, which could not be assessed by ITF, bound with moderate affinity (*K*_D_ = 15.1 ± 1.5 μM and 45.8 ± 7.9 μM, respectively). The detergents with the weakest affinity to hCD1d, as determined by MST, were CHAPS (~200 μM), LDAO (156.8 ± 58.6 μM), and ODG (6579.7 ± 186.0 μM). Some detergents, such as SDS and Deoxycholate, exhibited observable binding to CD1d, but *K*_D_ values could not be determined within the tested concentration range due to limited detergent solubility. Overall, the MST data corroborate the ITF assay and reveal pronounced differences in apparent detergent binding to hCD1d with Tyloxapol, Tween 20, Tween 80, and Brij‐35 as the strongest binders relative to SDS and ODG as the weakest binders.

### 2.3 NanoDSF reveals differences in detergent‐induced destabilization of hCD1d

**FIGURE 4:**
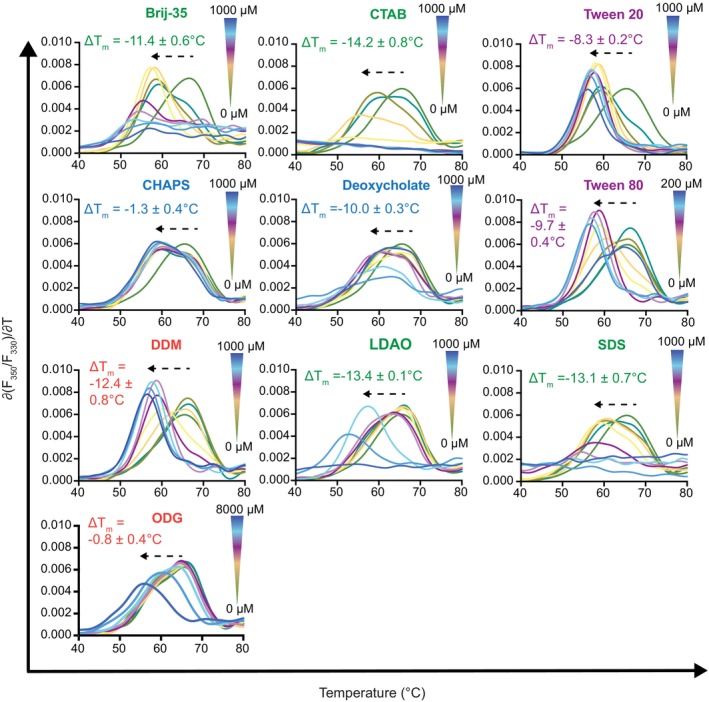
Measurements of detergent binding to hCD1d with nanoDSF. NanoDSF data showing the change in the first derivative of the Trp fluorescence ratio, ∂(*F*_350_/*F*_330_)/∂*T*, as a function of increasing concentrations of detergents with 1 μM hCD1d. The color gradient denotes the concentration range of the detergent tested, where the DSF spectra are color‐coded to match. The dotted arrows highlight the detergent‐dependent decrease in the melting temperature (*T*_m_) of hCD1d. Δ*T*_m_ is defined as the *T*_m_ difference between hCD1d in the absence and presence of 1000 μM detergent. Each data point is mean ± standard deviation for three replicates. The error in *K*_D_ is derived from the mean ± standard deviation of the individual fits of the three replicates. Detergent names are colored by class, as in Figure 1.

We next sought to probe the ability of each detergent to influence the thermal stability of hCD1d. NanoDSF has been previously applied to measure lipid/detergent‐induced thermal stability changes in target proteins (Cecchetti et al., 2021; Kotov et al., 2019), but has not yet been applied to ligand binding to CD1. NanoDSF measures the ITF ratio (*F*_350_ nm/*F*_330_ nm) of proteins as a function of temperature to extract thermodynamic parameters. Given that ligand binding typically shifts the melting temperature of proteins, the effect of a ligand on protein stability can be approximated with the Δ*T*_m_ value (the difference in *T*_m_ with and without excess ligand) (Cecchetti et al., 2021; Kotov et al., 2019). We found all detergents tested to destabilize hCD1d, albeit to different degrees, with Δ*T*_m_ values ranging from −0.8 to −14.2°C (Figures 4, S4, and Table 1). The detergents that destabilized hCD1d the most were CTAB (−14.2 ± 0.8°C), Deoxycholate (−10.0 ± 0.3°C), Brij‐35 (−11.4 ± 0.6°C), DDM (−12.4 ± 0.8°C), LDAO (−13.4 ± 0.1°C), and SDS (−13.1 ± 0.7°C). As highlighted by the disappearance of a clear inflection point in the first derivative of the Trp fluorescence ratio, ∂(*F*_350_/*F*_330_)/∂*T*, some detergents, such as Brij‐35, CTAB, Deoxycholate, LDAO, and SDS, appeared to unfold hCD1d at high detergent concentration (>200 μM). The detergents with the least ability to destabilize hCD1d, as determined by nanoDSF, were ODG (−0.8 ± 0.4°C) and CHAPS (−1.3 ± 0.4°C). As with ITF, only 10 of the 13 detergents could be evaluated by nanoDSF for the ability to destabilize hCD1d; Tyloxapol, NP‐40, and Triton X‐100 could not be probed due to their intrinsic fluorescence in the Trp fluorescence range.

**FIGURE 5:**
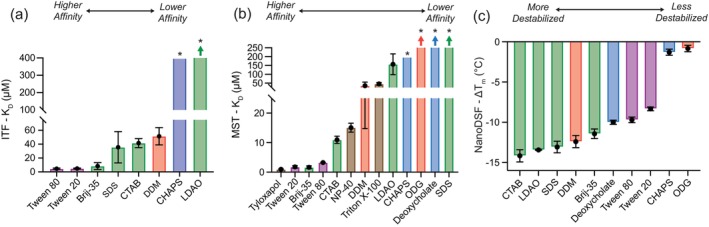
Comparison of detergent‐dependent binding affinity and destabilization effects on hCD1d. (a) Plot of ITF determined *K*_D_ values of different detergents with hCD1d. (b) Plot of MST determined *K*_D_ values of different detergents with hCD1d. (c) Plot of nanoDSF determined Δ*T*_m_ values with hCD1d. Δ*T*_m_ is defined as the *T*_m_ difference between hCD1d in the absence and presence of 1000 μM detergent. Each data point is mean ± standard deviation for three replicates. Each detergent name is colored by class: Sterol‐like detergents in blue, aromatic detergents in brown, glycosylated detergents in red, PEGylated sorbitols in purple, and aliphatic detergents in green. In panels (a) and (b), the asterisk represents estimated *K*_D_ values, and the arrows represent large *K*_D_ values outside of the plotting range (Table 1).

The results from ITF, MST, and nanoDSF collectively revealed distinct effects for different detergent classes (Figure 5). Sterol‐like and glycosylated detergents (CHAPS, ODG) exhibited weak binding (*K*_D_≈50–500 μM) and therefore minimal destabilizing effects (Δ*T*_m_≈−1 to −2°C). In contrast, PEGylated sorbitols and polymeric detergents (Tween 20, Tween 80, Brij‐35) were among the best binders (*K*_D_≈1–3 μM) and induced hCD1d destabilization to a moderate extent (Δ*T*_m_≈−8 to −10°C). Aliphatic detergents (CTAB, LDAO, SDS) showed intermediate binding affinity (*K*_D_≈10–500 μM) but caused significant hCD1d destabilization (Δ*T*_m_≈−12 to −15°C). Aromatic detergents (Tyloxapol, NP‐40, Triton X‐100) bound with moderate to high affinity (*K*_D_≈1–50 μM); their ability to influence hCD1d thermostability could not be assessed with nanoDSF. These findings highlight the importance of balancing binding strength with destabilizing effects when selecting detergents for CD1 applications.

### 2.4 In silico modeling suggests detergents bind in the hCD1d antigen‐binding groove

To glean insight into the mode of detergent binding to and destabilization of hCD1d, we generated detergent/hCD1d models using physics‐based docking software AutoDock Vina and the machine learning‐based structure prediction tool Chai‐1 (Discovery et al., 2024; Trott & Olson, 2010). AutoDock has previously been used to dock small molecules and lipids onto CD1 (Paterson et al., 2021; Rathakrishnan & McShan, 2023). Both rigid and flexible docking resulted in detergent poses within the CD1d binding groove; however, AutoDock Vina predicted binding affinity did not correlate with MST‐measured *K*_D_ values (Figure S5). As an alternative approach, we first evaluated the ability of Chai‐1 to model lipid/hCD1d complexes with available experimental structures. Chai‐1 was able to generate high‐confidence models of *α*‐GalCer/hCD1d (PDB ID 1ZT4) and 18:1 LPC/hCD1d (PDB ID 3U0P), which matched x‐ray structures with RMSD <1 Å (Figure S6).

**FIGURE 6:**
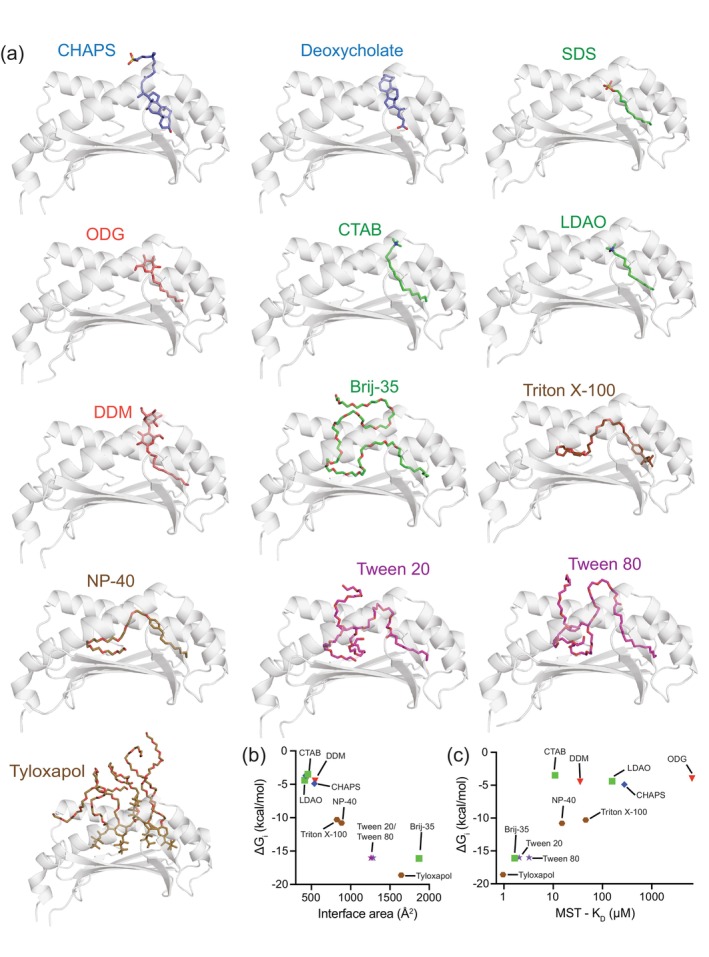
Chai‐1 models of detergents within the hCD1d binding groove. (a) The highest confidence Chai‐1 models (based on aggregate score and ipTM score) are shown. CD1d is shown as a cartoon colored gray. The CD1 *α*3 domain and *β*2m are not shown for clarity. Each detergent is colored by class: Sterol‐like detergents in blue, aromatic detergents in brown, glycosylated detergents in red, PEGylated sorbitols in purple, and aliphatic detergents in green. (b) PDBePISA determined interface Δ*G* (Δ*G*_*i*_) versus interface area between the detergent and hCD1d groove for each Chai‐1 model. (c) PDBePISA determined interface Δ*G* versus MST determined *K*_D_ values.

Chai‐1 models of detergents with hCD1d showed generally high confidence with interface predicted TM‐Score (ipTM) scores from 0.76 to 0.94, and pTM scores from 0.8 to 0.94 (Table S1). All detergents were predicted to dock into the hydrophobic hCD1d antigen binding groove, anchored primarily by aromatic and aliphatic side‐chains (Figures 6a and S7). Using the Chai‐1 models as input, interface free energy (Δ*G*_*i*_) and buried interface area between the detergent and the hCD1d groove were quantified with PDBePISA (Krissinel & Henrick, 2007) (Figure 6b). PEGylated sorbitols (Tween 20, Tween 80) appeared to cover larger surface areas with favorable Δ*G*_*i*_ values. Tyloxapol and Brij‐35 exhibited the largest interface areas and most favorable Δ*G*_*i*_. CTAB and LDAO showed the smallest interface areas with less favorable binding energetics. The PDBePISA determined Δ*G*_*i*_ values were also consistent with MST‐measured *K*_D_ values (Figure 6c). Tween 20, Tween 80, Tyloxapol, and Brij‐35 cluster at low *K*_D_ values and favorable Δ*G*, indicating both computational and experimental support for strong binding. In contrast, LDAO, ODG, and CHAPS had high *K*_D_ values and less favorable Δ*G*, supporting weaker interactions. Chai‐1 modeling was also able to provide a putative rationale for the striking difference between the observed binding affinities and protein destabilization exhibited by the n‐octyl *β*‐linked monosaccharide ODG (weak binding) and the n‐decyl *β*‐linked disaccharide DDM (moderate binding, strongly destabilizing). While ODG and DDM both were modeled to bind in the F′ pocket of hCD1d, DDM uniquely exhibited (i) engagement of two glucosyl moieties with the side‐chain groups of hCD1d, and (ii) additional Van der Waals contacts with the slightly longer nature of the alkyl group (C10 vs. C8), where the combination increased the buried surface area of DDM with CD1d (Figures 6a and S7).

Across all Chai‐1 models of detergent/hCD1d complexes, the most conserved hCD1d residues predicted to anchor the detergents into the antigen binding groove via hydrophobic and hydrogen bonding interactions were F77 (10/13 models), L148 (10/13 models), L96 (9/13 models), S76 (8/13 models), D80 (8/13 models), and L90/F84/W131/W140 (7/13 models) (Figure S7 and Table S2). We next asked whether residues that anchor detergents into the hCD1d groove in the Chai‐1 models were consistent with residues known to be involved in lipid antigen binding. The prototypical lipid antigen *α*‐GalCer (PDB ID 1ZT4) binds deeply in the hCD1d groove, anchored by 19 different residues forming hydrogen bonds and hydrophobic interactions commonly found to anchor lipids into the CD1d A′ and F′ pockets (Koch et al., 2005; López‐Sagaseta et al., 2012; Luoma et al., 2013; Paterson et al., 2021). Interestingly, we found that several hCD1d residues (Y73, S76, F84, L90, V116, L124, Y154) were strongly to moderately conserved across detergent/hCD1d models while also being involved in *α*‐GalCer binding, suggesting functional overlap between detergent and lipid antigen binding (Figure S7 and Table S2).

Given that some studies have reported species‐dependent observations of detergent binding to CD1 (Paletta et al., 2015), we also generated high‐confidence Chai‐1 models for detergent binding to CD1d orthologs from mouse, rat, rhesus monkey, pig, and cattle (Table S1). An amino acid sequence alignment between the orthologs highlights the presence of both conserved and non‐conserved residues in the antigen‐binding groove at sites predicted to be important for detergent binding (Figure S8). For some detergents, such as DDM, NP‐40, and CHAPS, Chai‐1 predicted binding poses were mostly consistent across the different CD1d orthologs (Figure S9). Other larger detergents, such as Brij‐35, Tween 20, and Tyloxapol, showed a wide range of binding poses across orthologs (Figure S9). These models suggest that different orthologs may support distinct detergent‐binding modes, destabilizing effects, or capacities for lipid‐antigen unloading, although this remains to be tested experimentally.

Together, the Chai‐1 models reveal a correspondence between predicted interface energetics, docking modes, and MST‐derived binding affinities, particularly for detergents like PEGylated sorbitols (Tween 20, Tween 80), Tyloxapol, and Brij‐35, which exhibit extensive interface areas and favorable binding free energies. Additionally, the residues predicted to mediate detergent binding frequently overlap with those involved in α‐GalCer binding, suggesting shared anchoring mechanisms within the hCD1d groove. Despite this agreement, some variability in predicted versus measured affinities points to the nuanced nature of detergent–protein interactions and underscores the importance of integrating computational and experimental approaches for characterization.

### 2.5 Comparison of detergent binding to a peptide/HLA complex

To determine whether detergent effects were specific to hCD1d, we performed analogous ITF, MST, and nanoDSF experiments with a peptide/HLA complex. HLA‐B*08:01, presenting the *Plasmodium falciparum* derived peptide CSP_19‐328_ (YLNKIQNSL), was selected as a representative model system since the peptide antigen contains hydrophilic solvent‐exposed residues in the antigen‐binding groove (Frooman et al., 2025). Although structurally similar to that of hCD1d, HLA‐B*08:01 shares low sequence identity (Figure S10a). We evaluated the ability of two detergents, ODG (low affinity for hCD1d) and Tween 20 (high affinity for hCD1d), to bind to either peptide/HLA or endogenous lipid/hCD1d complexes. By ITF, ODG produced minimal concentration‐dependent fluorescence changes for either complex (Figure S10b). In contrast, Tween 20 induced robust fluorescence changes for both peptide/HLA and lipid/hCD1d, yielding fitted K_D_ values of 7.7 ± 0.8 μM and 4.9 ± 0.7 μM, respectively (Figure S10c). By MST, ODG produced measurable but weak binding, with fitted K_D_ values of 3973.6 ± 289.7 μM for peptide/HLA and 6579.7 ± 186.0 μM for lipid/hCD1d (Figure S10d). Tween 20, in contrast, showed much stronger interactions, with *K*_D_ values of 10.1 ± 2.8 μM and 1.8 ± 0.5 μM for peptide/HLA and lipid/hCD1d, respectively (Figure S10e). By nanoDSF, ODG caused minimal destabilization of either complex (Δ*T*_m_≈−0.2 to −0.8°C; Figure S10f). Tween 20, however, exhibited a striking difference between the two antigen‐presenting molecules: it strongly destabilized lipid/hCD1d (Δ*T*_m_≈−8.3°C) but had only a minor effect on peptide/HLA (Δ*T*_m_≈−0.3°C; Figure S10g). Together, these data indicate that both ODG and Tween 20 have the potential to associate with peptide/HLA and lipid/hCD1 complexes to varying degrees. However, their effects, particularly the strong destabilization of hCD1d by Tween 20, are not conserved across antigen‐presenting molecules and likely reflect specific detergent‐mediated unloading of endogenous lipids from hCD1d. We posit that lipid‐specific unloading effects of detergent are due to either hydrophobic residues in the antigen binding groove (detergent‐CD1d interactions) and/or the hydrophobic nature of the lipid antigen (detergent‐lipid interactions).

### 2.6 High‐affinity detergents efficiently block the binding of 18:1 LPC, but not *α*‐GalCer, to hCD1d

**FIGURE 7:**
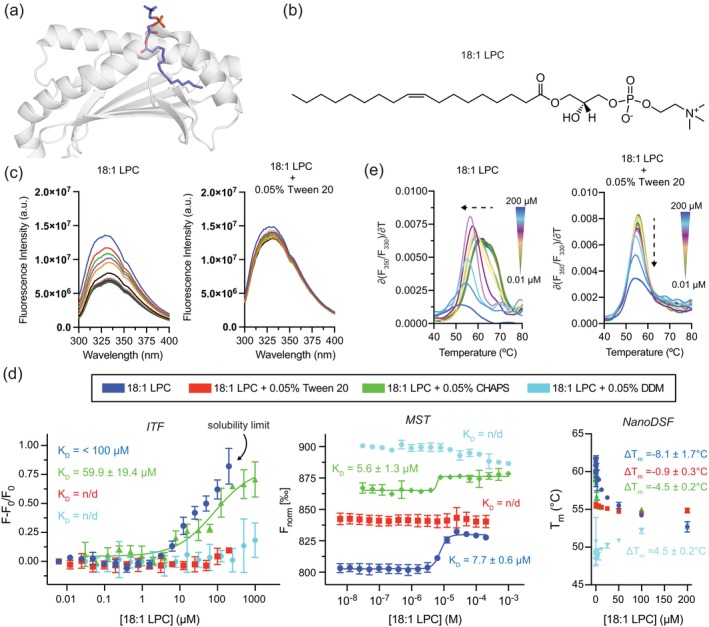
High‐affinity detergents block binding of 18:1 LPC to hCD1d. (a) Side view of the 18:1 LPC/hCD1d complex. CD1d is shown as a cartoon colored gray. The LPC lipid is colored as blue sticks. The x‐ray structure was taken from PDB ID 3U0P (López‐Sagaseta et al., 2012). (b) Chemical structure of 18:1 LPC. (c) Left: Raw ITF spectra of increasing concentrations of 18:1 LPC in the presence of 100 nM hCD1d in the absence and presence of 0.05% Tween 20 at 25°C. (d) Left: ITF data showing the change in Trp fluorescence (*F* − *F*_0_/*F*_0_ where *F*_0_ is initial fluorescence in the absence of 18:1 LPC) as a function of increasing concentrations of 18:1 LPC in the presence of 100 nM hCD1d without or with 0.05% Tween 20, CHAPS, or DDM at 25°C. Fits of the binding isotherms are as shown. Middle: MST data showing the change in normalized fluorescence signal as a function of increasing concentrations of 18:1 LPC with 100 nM AF647‐labeled hCD1d without or with 0.05% Tween 20, CHAPS, or DDM at 25°C. Right: The nanoDSF determined melting temperature (*T*_m_) of hCD1d as a function of increasing concentrations of 18:1 LPC (μM) without or with 0.05% Tween 20, CHAPS, or DDM. Δ*T*_m_ is defined as the *T*_m_ difference between CD1d in the absence and presence of 200 μM 18:1 LPC. (e) NanoDSF data showing the change in the first derivative of the Trp fluorescence ratio, ∂(*F*_350_/*F*_330_)/∂*T*, as a function of increasing concentrations of 18:1 LPC with 1 μM hCD1d without or with 0.05% Tween 20. The color gradient denotes the concentration range of 18:1 LPC, where the DSF spectra are color‐coded. The dotted arrows highlight the direction of change in the *T*_m_ of hCD1d. Each data is mean ± standard deviation for three replicates.

To investigate the effect of detergent on lipid/hCD1d binding, we evaluated the interaction of 18:1 lysophosphatidylcholine (18:1 LPC) with hCD1d using ITF, MST, and nanoDSF in the absence and presence of Tween 20 (Figure 7a,b). We selected 18:1 LPC for the competition experiments since, first, a structure with hCD1d has been well characterized, and second, it binds hCD1d with moderate micromolar affinity, which is representative of many endogenous lipid antigens (Cantu et al., 2003; López‐Sagaseta et al., 2012). In the absence of detergent, 18:1 LPC produced concentration‐dependent changes in ITF and MST signal and decreased the melting temperature of hCD1d (Δ*T*_m_ = −8.1 ± 1.7°C), indicative of specific binding and hCD1d destabilization (Figure 7c–e). Fitted *K*_D_ values (approx. 35.6 ± 14.4 μM by ITF and 7.7 ± 0.6 μM by MST) are consistent with the μM affinity range of 18:1 LPC for hCD1d previously measured by ITF (López‐Sagaseta et al., 2012). The addition of high‐affinity detergent Tween 20 or moderate‐affinity detergent DDM abolished nearly all detectable binding signals between 18:1 LPC and hCD1d across ITF, MST, and nanoDSF experiments (Figure 7c–e). This is consistent with the low‐micromolar binding constants of the detergents, which presumably allow for direct inhibition of 18:1 LPC interaction with hCD1d through steric competition for the overlapping binding sites in the hCD1d groove (Figures 6 and 7). In contrast, low‐affinity detergent CHAPS did not robustly outcompete binding of 18:1 LPC with hCD1d (Figure 7d), providing evidence that lipid/detergent competition for hCD1d depends on detergent affinity for the antigen binding groove.

To determine whether Tween 20 universally interferes with lipid/hCD1d interactions, even for high‐affinity lipid antigens, we assessed the binding of the well‐characterized glycolipid antigen α‐GalCer to hCD1d (Cantu et al., 2003; Kobayashi et al., 1995; Koch et al., 2005; Tsuji, 2006). The binding affinity of *α*‐GalCer to hCD1d has been previously estimated to be ~0.1 to 0.5 μM (Cantu et al., 2003; Naidenko et al., 1999), although these experiments were performed in the presence of detergent. Unfortunately, we could not measure the binding of *α*‐GalCer to hCD1d in the absence of Tween 20 due to the limited solubility of *α*‐GalCer. However, unlike 18:1 LPC, the addition of *α*‐GalCer produced a marked increase in thermal stabilization of hCD1d (Δ*T*_m_ = +13.7 ± 0.2°C) and a measurable MST response with an estimated *K*_D_ of 6.6 ± 4.1 μM, even in the presence of 0.05% Tween 20 (Figure S11a–c). We attempted to perform analogous competition experiments for *α*‐GalCer to hCD1d with DDM and CHAPS, but *α*‐GalCer was only soluble in the presence of Tween 20. These findings highlight the importance of acknowledging the presence and role of detergents in working with CD1 proteins. In this case, for example, the binding constant of *α*‐GalCer to hCD1d is very likely much lower (greater affinity) than the 0.1–0.5 μM value reported in the literature, which should more properly be referred to as a competitive, rather than absolute, binding constant and include the identity (and concentration, if possible) of any detergent additive present.

Taken together, these findings highlight the importance of experimental conditions in studying lipid/hCD1d interactions and suggest that detergents like Tween 20, Tween 80, DDM, Brij‐35, and Tyloxapol (Table 1) may selectively alter lipid binding events that affect canonical antigen presentation.

## 3 DISCUSSION

The integrated biophysical and computational analysis presented here provides compelling evidence that detergents interact with and destabilize hCD1d in a detergent‐specific manner. Consistent with previous reports on the potential of detergents to influence CD1‐dependent T cell staining or mass spectrometry‐based lipidomics efforts (Gadola et al., 2002; Im et al., 2009; Le Nours et al., 2016; Mansour et al., 2016; Uldrich et al., 2013), our data demonstrate that detergent molecules have the potential to directly occupy the hydrophobic CD1d groove, thereby functioning as competitive inhibitors of native lipid antigen binding.

One novel aspect of this work is the first application of MST and nanoDSF to probe detergent and lipid binding to CD1 molecules (Figures 3, 4, and 7). Together, these complementary assays provide an extended framework for assessing lipid/CD1 interactions across a broad range of detergents and lipids with unique chemistry and structure. When combined with existing lipid loading/exchange assays, immunological assays, and structural determination efforts, the combined suite of ITF, MST, and nanoDSF assays has the potential to enable the quantification of biophysical parameters underpinning lipid/CD1 interactions, aid the discovery of novel lipid antigens, and support the development of high‐affinity analogues for immunomodulation. MST and nanoDSF's relatively low sample requirements and high‐throughput nature make them particularly well suited for screening libraries of candidate lipid antigens or chemical probes for CD1. Even though ITF, MST, and nanoDSF assays report on distinct biophysical phenomena, they seem to provide both complementary and corroborating information on ligand/CD1 binding events (Figure 5). The determined K_D_ values for detergent binding were well below each detergent's CMC value, suggesting that ITF, MST, and nanoDSF report on specific detergent/hCD1d interactions rather than non‐specific micelle binding (Table 1).

The fine‐tuned balance between detergent class, size, chemistry, and ability to form interactions with residues in the hCD1d groove dictates specificity, affinity, and the potential to induce destabilization (Figures 1 and 5, and S7). Detergent‐specific affinities and hCD1d‐destabilizing effects suggest underlying differences in binding mechanisms. For instance, anionic detergents CTAB, LDAO, and SDS cause pronounced hCD1d destabilization, despite only moderate binding affinities (Figure 5), implying the presence of unfavorable electrostatic interactions or groove distortions. Conversely, weakly interacting detergents such as zwitterionic CHAPS or non‐ionic ODG had minimal effects on hCD1d stability (Figure 5), supporting their use as mild agents for CD1 structural or binding studies. The non‐ionic detergents Tween 20, Tween 80, Tyloxapol, and Brij‐35 exhibited the highest affinities and only moderately destabilized hCD1d, where Chai‐1 models predict anchoring hydrophobic interactions spanning both A′ and F′ pockets that support their role as “goldilocks” displacers of lipids in the binding groove (Figures 5 and S7). There are likely to be CD1 isoform‐dependent differences in detergent binding due to antigen‐binding groove chemistry and geometry. The ability of some detergents (i.e., Triton X‐100, Tween 20, CHAPS) to unload lipids from CD1 molecules has previously been demonstrated for CD1c (Mansour et al., 2016). Notably, many residues predicted to form Van der Waals or hydrogen bonding contacts with detergents are conserved across other CD1 isoforms (Figure S12), raising the possibility that similar detergent‐binding behavior may occur in those systems as well. However, isoform‐specific differences in groove architecture, pocket depth, and electrostatics may also produce unique interaction profiles. For example, groove volumes of the CD1 isoforms vary considerably from 1300 to 2200 Å^3^ (Schiefner & Wilson, 2009), which could alter the ability to accommodate detergents of different sizes. Future experiments should probe the ability of detergents to associate with CD1a, CD1b, and CD1c.

Our findings offer practical guidance for selecting detergents in biochemical workflows involving CD1 proteins, taking into account destabilizing and detergent‐binding effects. The observation that high‐affinity detergents, such as Tween 20, Tween 80, Tyloxapol, and Brij‐35, have the potential to effectively compete with endogenous or exogenous lipids for the CD1 groove underscores the need for careful assay design (Table 1 and Figure 7). In lipid‐loading or antigen exchange assays, inappropriate detergent selection may reduce loading efficiency or obscure tetramer staining data (Gadola et al., 2002; Im et al., 2009; Le Nours et al., 2016; Mansour et al., 2016; Uldrich et al., 2013). High‐affinity lipids, such as *α*‐GalCer, may overcome this competition to some extent, but the general principle remains: detergents can significantly interfere with lipid binding to the CD1 groove. This also has additional implications for lipidomics pipelines in which detergents are used during protein extraction or purification; such protocols risk displacing physiologically relevant lipids before mass spectrometry identification (Gadola et al., 2002; Im et al., 2009; Le Nours et al., 2016; Mansour et al., 2016; Uldrich et al., 2013).

This work also has several limitations. First, the biophysical assays conducted here in reality reflect competition between exogenous detergents/lipids and the pool of endogenous lipids that co‐purify with hCD1d expressed in mammalian expression systems (Huang et al., 2023). This complicates quantitative interpretation of “apparent” *K*_D_ values, as detergent binding may involve partial or complete displacement of endogenous ligands. Second, although it cannot be definitively ruled out that detergents may bind at alternative sites on the CD1 complex, such as the CD1 *α*3 domain or *β*2m interface, multiple lines of evidence strongly support the primary binding site within the antigen‐binding groove. These include the observed detergent/lipid competition in MST, ITF, and nanoDSF assays (Figures 7 and S11), as well as in silico modeling results with Chai‐1 that place detergents within the canonical lipid‐binding pockets of hCD1d (Figures 6 and S7). Additional structural and mutagenesis studies will be required to experimentally confirm the predicted detergent binding modes. Finally, saturating ITF and MST binding curves were difficult for some lipids and detergents because of solubility limits and CD1 destabilization at higher detergent concentrations, an issue likely shared by many endogenous lipids.

In summary, this study reveals that detergents are not biologically inert components in CD1 workflows, but active ligands capable of modulating protein conformation and stability. Thus, they have the potential to compete with natural lipid or small molecule antigens. MST and nanoDSF provide sensitive, orthogonal platforms to dissect these interactions and offer a path forward for systematic screening of lipid/CD1 binding in a detergent‐sensitive context. These insights will be important for refining lipid‐loading protocols, lipidomics workflows, and designing new immunomodulatory lipids.

## 4 MATERIALS AND METHODS

### 4.1 Detergents

The following 13 detergents were purchased: Tween 20 (Sigma‐Aldrich #P7949), Tween 80 (Fisher Scientific #BP338), n‐octyl‐*β*‐D‐glucopyranoside (ODG, Santa Cruz Biotechnology #sc‐281,091), Triton X‐100 (Sigma‐Aldrich #X100), n‐dodecyl‐*β*‐D‐maltoside (DDM, GoldBio #DDM5), Tyloxapol (Sigma‐Aldrich #T8761), NP‐40 (Thermo Scientific #28324), Brij‐35 (Thermo Scientific #20150), hexadecyltrimethylammonium bromide (CTAB, Sigma‐Aldrich #H5882), sodium deoxycholate (Sigma‐Aldrich #D6750), sodium dodecyl sulfate (SDS, TCI #D1403), lauryldimethylamine‐N‐oxide (LDAO, Thermo Scientific #A65503), and 3‐((3‐cholamidopropyl) dimethylammonio)‐1‐propanesulfonate (CHAPS, Fisher Scientific #BP571). Detergents were resuspended to desired stock concentrations in 1X PBS pH 7.4 using sonication/heating. The following lipids were purchased: 1‐oleoyl‐2‐hydroxy‐sn‐glycero‐3‐phosphocholine (18:1 LPC, Avanti Research #845875) and α‐galactosyl ceramide (*α*‐GalCer, Avanti Research #867000). For resuspension, 18:1 LPC was resuspended in 1X PBS pH 7.4, and α‐GalCer was suspended in 1X PBS pH 7.4, 0.05% Tween 20 using sonication/heating.

### 4.2 Recombinant expression and purification of schCD1d

A soluble single‐chain construct of human CD1d (schCD1d, herein called hCD1d) was prepared as described previously (Im et al., 2004). Briefly, the construct contained (from N‐ to C‐terminus): human *β*2 microglobulin (*β*2M, residues 1–119, UniProt #P61769), a glycine/serine linker (GGGSGGSGSGGGA), human CD1d ectodomain (residues 24–295, UniProt #P15813), a TEV protease cleavage site (ENLYFQS), a glycine/serine linker (GS), a BirA biotinylation site (HVGLNDIFEAQKIEWHEGH), and a His_7_ tag for affinity purification (HHHHHHH).

The resulting protein sequence (prior to in vivo cleavage of the *β*2m signal peptide):


MSRSVALAVLALLSLSGLEAIQRTPKIQVYSRHPAENGKSNFLNCYVSGFHPSDIEVDLLKNGERIEKVEHSDLSFSKDWSFYLLYYTEFTPTEKDEYACRVNHVTLSQPKIVKWDRDMGGGSGGSGSGGGARLFPLRCLQISSFANSSWTRTDGLAWLGELQTHSWSNDSDTVRSLKPWSQGTFSDQQWETLQHIFRVYRSSFTRDVKEFAKMLRLSYPLELQVSAGCEVHPGNASNNFFHVAFQGKDILSFQGTSWEPTQEAPLWVNLAIQVLNQDKWTRETVQWLLNGTCPQFVSGLLESGKSELKKQVKPKAWLSRGPSPGPGRLLLVCHVSGFYPKPVWVKWMRGEQEQQGTQPGDILPNADETWYLRATLDVVAGEAAGLSCRVKHSSLEGQDIVLYWENLYFQSGSHVGLNDIFEAQKIEWHEGHHHHHHH





A codon‐optimized synthetic DNA encoding the above sequence and an upstream Kozak sequence (CCGCCGCCACC) was cloned into the pcDNA3.4 vector with EcoRI/HindIII restriction sites and expressed in TurboCHO cells (GenScript). The supernatant was purified by nickel‐NTA affinity chromatography (HisTrap FF 5 mL, Cytiva) at a flow rate of 5 mL/min using an ÄKTA go FPLC system. Following loading of the sample, the column was washed with 5 column volumes of Buffer A (300 mM NaCl, 50 mM Tris, 10 mM imidazole buffer, pH 8). Protein was eluted using a linear gradient of Buffer B (300 mM NaCl, 50 mM Tris, 500 mM imidazole, pH 8) from 0 to 100% over 10 column volumes. The fractions containing design proteins were then concentrated to 500 μL with 10 kDa Amicon Ultra Centrifugal Filters. Proteins were further purified by size‐exchange chromatography (SEC) with a Superdex 200 Increase 10/300 GL column at a flow rate of 0.5 mL/min in SEC buffer (1X PBS pH 7.4). Protein purity was confirmed with SDS‐PAGE. Protein concentration was measured using a NanoDrop with extinction coefficients (A_280_ nm) calculated with ExPASy ProtParam (Gasteiger et al., 2003).

### 4.3 Recombinant expression and purification of peptide/HLA molecules

For comparison with peptide/HLA molecules, we prepared recombinant CSP 319‐328/HLA‐B*08:01/h*β*2M complex using in vitro refolding methods fully described previously (Frooman et al., 2025). The peptide sequence for CSP 319–328 is YLNKIQNSL. This peptide was chosen since it contains Lys, Gln, and Asp residues predicted to be solvent‐exposed in the HLA‐B*08:01 binding groove (Frooman et al., 2025).

### 4.4 Circular dichroism spectroscopy (CD)

Far‐UV CD spectra were collected on a JASCO J‐815 Spectropolarimeter. CD spectra were measured from 190 to 350 nm with 0.35 mg/mL hCD1 in CD buffer [50 mM NaCl, 20 mM sodium phosphate, pH 7.2] using 1 mm path length quartz cuvettes (Starna Cells #21‐Q‐1/CD). CD spectra were acquired a total of 10 times at 25°C with a scan rate of 50 nm/min and then averaged. The average CD spectra were smoothed using a Savitzky–Golay filter with a convolution width of 9.

### 4.5 Intrinsic tryptophan fluorescence (ITF)

ITF spectra were recorded from 300 to 500 nm using a Horiba FL3‐21 Fluorometer with the excitation wavelength of 285 nm and excitation/emission slits of 6.2 nm. Fluorescence spectra of 50 to 100 nM hCD1d were acquired in the absence and presence of detergents in 1X PBS pH 7.4 at 25°C. Prior to data acquisition, hCD1d and detergent or lipid samples were incubated at 37°C overnight (~12 h) to simulate in vitro and in vivo loading conditions. For most detergents, serial dilutions of the detergents resulted in testing 16 concentrations (in μM): 1000, 500, 250, 125, 62.5, 31.25, 15.62, 7.81, 3.90, 1.95, 0.97, 0.48, 0.24, 0.12, 0.06, and 0.03. For ODG experiments, serial dilutions of the detergents resulted in testing 16 concentrations (in mM): 32, 16, 8, 4, 2, 1, 0.5, 0.25, 0.12, 0.06, 0.03, 0.01, 0.007, 0.003, 0.001, and 0.0009. For *α*‐GalCer experiments, fluorescence spectra of 100 nM hCD1d were acquired in the absence and presence of *α*‐GalCer in 1X PBS pH 7.4 containing 0.05% Tween 20 at 25°C. The concentration of 0.05% Tween 20 was consistent across all experiments. Serial dilutions of *α*‐GalCer resulted in testing 16 concentrations (in μM): 50, 25, 12.5, 6.25, 3.12, 1.56, 0.78, 0.39, 0.19, 0.09, 0.04, 0.02, 0.01, 0.006, 0.003, and 0.001. For 18:1 LPC experiments, fluorescence spectra of 100 nM hCD1d were acquired in the absence and presence of 18:1 LPC in 1X PBS pH 7.4 at 25°C. Experiments were performed with and without a buffer containing 0.05% Tween 20 in the buffer. Serial dilutions of 18:1 LPC resulted in testing 16 concentrations (in μM): 200, 100, 50, 25, 12.5, 6.25, 3.12, 1.56, 0.78, 0.39, 0.19, 0.09, 0.04, 0.02, 0.01, and 0.006. ITF experiments could not be performed for NP‐40, Triton X‐100, and Tyloxapol due to their intrinsic fluorescence signal that overlaps with tryptophan. For all ITF experiments, the change in fluorescence signal (*F* − *F*_0_/*F*_0_) was determined at 335 nm. Three replicates were performed, and data are presented as the mean ± standard deviation (*n* = 3). *K*_D_ values were fitted with non‐linear regression in GraphPad Prism v10.1.

ITF experiments with peptide/HLA molecules and detergents were performed using an analogous protocol.

### 4.6 Microscale thermophoresis (MST)

For MST, hCD1d was fluorescently labeled with AF647‐NHS Ester (Invitrogen #A20006). Briefly, a 10 mM stock of AF647‐NHS was prepared in anhydrous DMSO. Then, 100 μL of 50 μM hCD1d in 1X PBS pH 7.4 was mixed with 1 μL of 10 mM AF647‐NHS (1:2 molar ratio of protein: dye). The mixture was allowed to react for 1 h in the dark at 25°C. Unreacted dye was removed from the protein–dye conjugate by performing size exclusion chromatography in the dark with a Superdex 200 Increase 10/300 GL column at a flow rate of 0.5 mL/min in SEC buffer (100 mM NaCl, 20 mM sodium phosphate, pH 7.2). The degree of labeling was determined by a NanoDrop spectrophotometer at A_647_ nm (dye absorbance) and A_280_ nm (hCD1d absorbance). The degree of labeling was determined from the ratio of dye concentration to protein concentration. The degree of labeling was ~75%.

MST experiments were performed using a Monolith NT.115 instrument (NanoTemper Technologies) using Monolith standard capillaries and the red excitation laser setup. The following settings were applied for all MST experiments: excitation power: 30%–50%, MST power: medium, before MST time: 3 s, MST on time (IR laser): 20 seconds, and after MST time: 1 s. MST measurements were analyzed using the Monolith Affinity Analysis software v2.3 and exported for plotting in GraphPad Prism v10.1. Three replicates were performed, and data are presented as the mean ± standard deviation (*n* = 3).

MST measurements of 100 nM AF647‐hCD1d were acquired in the absence and presence of detergents in 1X PBS pH 7.4 at 25°C. Prior to data acquisition, hCD1d and detergent or lipid samples were incubated at 37°C overnight (~12 h) to simulate in vitro and in vivo loading conditions. For most detergents, serial dilutions of the detergents resulted in testing 16 concentrations (in μM): 1000, 500, 250, 125, 62.5, 31.25, 15.62, 7.81, 3.90, 1.95, 0.97, 0.48, 0.24, 0.12, 0.06, and 0.03. For ODG experiments: serial dilutions of the detergents resulted in testing 16 concentrations (in mM): 32, 16, 8, 4, 2, 1, 0.5, 0.25, 0.12, 0.06, 0.03, 0.01, 0.007, 0.003, 0.001, and 0.0009. For *α*‐GalCer experiments: MST data of 100 nM AF647‐hCD1d were acquired in the absence and presence of α‐GalCer in 1X PBS pH 7.4 containing 0.05% Tween 20 at 25°C. The concentration of 0.05% Tween 20 was consistent across all experiments. Serial dilutions of *α*‐GalCer resulted in testing 16 concentrations (in μM): 50, 25, 12.5, 6.25, 3.12, 1.56, 0.78, 0.39, 0.19, 0.09, 0.04, 0.02, 0.01, 0.006, 0.003, and 0.001. For 18:1 LPC experiments: MST data of 100 nM AF647‐hCD1d were acquired in the absence and presence of 18:1 LPC in 1X PBS pH 7.4 at 25°C. Experiments were performed with and without buffer containing 0.05% Tween 20, 0.05% CHAPS, or 0.05% DDM in the buffer. Serial dilutions of 18:1 LPC resulted in testing 16 concentrations (in μM): 200, 100, 50, 25, 12.5, 6.25, 3.12, 1.56, 0.78, 0.39, 0.19, 0.09, 0.04, 0.02, 0.01, and 0.006.

MST experiments with peptide/HLA molecules and detergents were performed using an analogous protocol.

### 4.7 Nano differential scanning fluorimetry (NanoDSF)

Nano differential scanning fluorometry (nanoDSF) was performed using a Prometheus NT.48 (NanoTemper Technologies). Purified hCD1d at 1 μM in 1X PBS pH 7.4 was loaded into nanoDSF grade standard capillaries (NanoTemper Technologies) and exposed to temperatures ranging from 20 to 95°C with a thermal ramping rate of 1°C/min. Excitation power was set to 50%. The ratio of intrinsic fluorescence emission (*F*_350_ nm/*F*_330_ nm) was monitored. Melting temperatures (*T*_m_) were obtained from fitting the first derivative plots in the Prometheus NT.48 Software. The experiments were performed in triplicate, and data are presented as the mean ± standard deviation (*n* = 3).

For experiments with most detergents, prior to data acquisition, hCD1d and detergent or lipid samples were incubated at 37°C overnight (~12 h) to simulate in vitro and in vivo loading conditions. Serial dilutions of the detergents resulted in testing 16 concentrations (in μM): 1000, 500, 250, 125, 62.5, 31.25, 15.62, 7.81, 3.90, 1.95, 0.97, 0.48, 0.24, 0.12, 0.06, and 0.03. For ODG experiments: serial dilutions of the detergents resulted in testing 16 concentrations (in mM): 32, 16, 8, 4, 2, 1, 0.5, 0.25, 0.12, 0.06, 0.03, 0.01, 0.007, 0.003, 0.001, and 0.0009. For *α*‐GalCer experiments: nanoDSF spectra were acquired in the absence and presence of *α*‐GalCer in 1X PBS pH 7.4 containing 0.05% Tween 20 at 25°C. The concentration of 0.05% Tween 20 was consistent across all experiments. Serial dilutions of *α*‐GalCer resulted in testing 16 concentrations (in μM): 50, 25, 12.5, 6.25, 3.12, 1.56, 0.78, 0.39, 0.19, 0.09, 0.04, 0.02, 0.01, 0.006, 0.003, and 0.001. For 18:1 LPC experiments: nanoDSF spectra were acquired in the absence and presence of 18:1 LPC in 1X PBS pH 7.4 at 25°C. Experiments were performed with and without a buffer containing 0.05% Tween 20 in the buffer. Serial dilutions of 18:1 LPC resulted in testing 16 concentrations (in μM): 200, 100, 50, 25, 12.5, 6.25, 3.12, 1.56, 0.78, 0.39, 0.19, 0.09, 0.04, 0.02, 0.01, and 0.006. nanoDSF experiments could not be performed for NP‐40, Triton X‐100, and Tyloxapol due to their intrinsic fluorescence signal that overlaps with tryptophan.

NanoDSF experiments with peptide/HLA molecules and detergents were performed using an analogous protocol.

### 4.8 Structure modeling and computational analysis

Detergent/CD1d/*β*2M complexes were modeled using the Chai‐1 webserver (https://lab.chaidiscovery.com/dashboard) (Discovery et al., 2024). Protein sequences and SMILES strings used as input are noted in Table S1. The “best” resulting model was chosen based on the overall Chai‐1 aggregate score (interface predicted TM‐Score), ipTM score, and pTM score. Detergent/hCD1d interactions were determined from the Chai‐1 models with the Protein‐Ligand Interaction Profiler (PLIP) tool v2.4.0 using default parameters (Salentin et al., 2015). To determine interface area and interface ΔG values between the hCD1d groove and detergents, the Chai‐1 models were submitted to PDBePISA (Proteins, Interfaces, Structures and Assemblies) (https://www.ebi.ac.uk/pdbe/pisa/) in interface mode (Krissinel & Henrick, 2007).

### 4.9 Molecular docking with AutoDock Vina

AutoDock Vina was used for in silico molecular docking to predict binding conformations of detergents within the hCD1d antigen binding groove (Trott & Olson, 2010). The x‐ray structure of human CD1d bound to ɑ‐galactosylceramide (PDB ID 1ZT4) was used (Koch et al., 2005). Atomic coordinates corresponding to water molecules and ɑ‐galactosylceramide were removed from the PDB file prior to docking, leaving only hCD1d atoms. The hCD1d structure was prepared using standard procedures in the AutoDock tools suite (Forli et al., 2016). Two different docking protocols were performed: one where all hCD1d residues were considered rigid, and one where hCD1d residues in the antigen binding groove (positions 10, 12, 14, 16, 30, 32, 38, 63, 66, 70, 73, 77, 81, 84, 96, 98, 100, 112, 114, 116, 123, 124, 126, 131, 148, 154, 158, and 169 corresponding to numbering for PDB ID 1ZT4) were allowed to be flexible. The search space for molecular docking was defined on the CD1d receptor to configure AutoDock Vina to only dock detergents within the antigen‐binding groove. Default settings of AutoDock Vina were used with the exhaustiveness parameter set to 8. The 3D conformers SDF file for detergents was obtained from PubChem. 3D SDF files were converted into 3D coordinate PDBQT files using OpenBabel v3.1.0 for compatibility with AutoDock Vina (O'Boyle et al., 2011). The binding pose with the lowest AutoDock predicted affinity (kcal/mol) for each CD1d‐detergent complex was selected as the most likely predicted conformation.

## AUTHOR CONTRIBUTIONS

**Uri Z. Miles:** Investigation; writing – review and editing; conceptualization; formal analysis; methodology. **M. G. Finn:** Writing – review and editing; supervision. **Andrew C. McShan:** Conceptualization; investigation; funding acquisition; writing – original draft; methodology; writing – review and editing; project administration; supervision; formal analysis; visualization.

## CONFLICT OF INTEREST STATEMENT

The authors declare that no competing interests exist.

## Supporting information


**Supplementary Figure S1.** Purification and characterization of a recombinant single‐chain construct of hCD1d. (A) Following a Ni‐NTA affinity column, the second round purification of size exclusion chromatography purification of hCD1d with a Superdex 200 Increase 10/300 GL column at 0.5 mL/min in 1X PBS pH 7.4. (B) SDS‐PAGE of purified hCD1d with PageRuler Unstained Protein Ladder (Thermo Fisher Scientific #26614). The protein travels higher than its expected molecular weight (~47.5 kDa) due to glycosylation from mammalian cell expression. (C) Far‐UV circular dichroism spectra of 0.35 mg/mL hCD1d recorded at 25°C recorded in the absence of detergent. The characteristic negative band near 218 nm corresponds to *β*‐sheet secondary structure, which is the main component of the immunoglobulin fold of CD1d and *β*2m. The CD spectra profile is also consistent with previously acquired CD spectra of CD1 molecules. (D) First derivative of the nanoDSF spectra (*F*_350_/*F*_330_) of 1 μM hCD1d recorded in the absence of detergent. The fitted melting temperature (*T*_m_) is noted.
**Supplementary Figure S2.** Raw ITF data. Fluorescence spectra of tryptophan from 100 nM hCD1d in the absence and presence of increasing amounts of detergents acquired at 25°C.
**Supplementary Figure S3.** Raw MST data. Raw MST traces of 100 nM AF647‐hCD1d in the absence and presence of increasing amounts of detergents acquired at medium MST power at 25°C with LED power set between 30% and 50%.
**Supplementary Figure S4.** Determination of Δ*T*_m_ values from nanoDSF data. Each plot shows the nanoDSF determined melting temperature (*T*_m_) of hCD1d as a function of increasing concentrations of detergents (μM). For each detergent concentration, the schCD1d *T*_m_ was determined from the inflection point of the first derivative curve of the Trp fluorescence ratio, ∂(*F*_350_/*F*_330_)/∂*T*. The red arrows highlight the detergent concentration at which the ∂(*F*_350_/*F*_330_)/∂*T* goes into the noise. Each data point is the mean ± standard deviation for three replicates.
**Supplementary Figure S5.** Modeling detergent binding poses in the hCD1d antigen binding groove with AutoDock Vina. Top: Overlay of detergent binding poses obtained from Chai‐1 (green sticks), rigid AutoDock Vina (magenta sticks), and flexible AutoDock Vina (marine sticks). Bottom: AutoDock Vina predicted detergent affinity (kcal/mol) from AutoDock Vina binding poses versus MST determined *K*_D_ values compared with PDBePISA determined interface Δ*G* from Chai‐1 binding poses versus MST determined *K*_D_ values.
**Supplementary Figure S6.** Comparison of in silico Chai‐1 models with x‐ray structures for native lipid antigens with hCD1d. Overlay of Chai‐1 models (green) versus x‐ray structures (cyan) for *α*‐GalCer/hCD1d (PDB ID 1ZT4) and 18:1 LPC/hCD1d (PDB ID 3U0P). Lipid atoms and CD1d residues that interact with the lipid antigens are shown as sticks. The CD1d backbone is shown as a cartoon. Chai‐1 model confidence (ipTM, pTM) and heavy chain RMSD values (determined by PyMOL v3.1.6.1) are noted.
**Supplementary Figure S7.** Details of detergent/hCD1d interactions from in silico Chai‐1 models. Molecular interactions between detergents and hCD1d groove from the Chai‐1 models. Hydrogen bonds (blue dashes), hydrophobic interactions (black dashes), and salt bridges (yellow dashes) are predicted by the PLIP tool (Salentin et al., 2015). Models are oriented as shown in Figure 6. CD1d residues are shown as dark blue sticks; the CD1d backbone cartoon is not shown for clarity.
**Supplementary Figure S8.** Comparison of predicted detergent binding residues across the antigen binding groove residues of CD1d orthologs. Protein sequence alignment was performed for the ectodomain of CD1d molecules with Clustal Omega v1.2.4 and visualized in ESPript v3.0. Sequences used: *Homo sapiens* CD1d (human, UniProt #P15813), *Mus musculus* (mouse, UniProt #P11609), *Rattus norvegicus* (rat, UniProt #Q63493), *Macaca mulatta* (rhesus macaque, UniProt #F6TR81), *Sus scrofa* (pig, UniProt #A0ZQ05), and *Bos taurus* (bovine, UniProt #A1L565). The blue triangles represent CD1d residues predicted to interact with detergents based on Chai‐1 models (see Table S2).
**Supplementary Figure S9.** Chai‐1 modes of CD1d orthologs with different detergents.
**Supplementary Figure S10.** Binding of ODG and Tween 20 to a peptide/HLA complex by ITF, MST, and nanoDSF. (A) Sequence alignment for the antigen‐binding groove residues of the ectodomain of human HLA‐B*08:01 (UniProt #P01889) and human CD1d (UniProt #P15813) with Clustal Omega v1.2.4 and visualized in ESPript v3.0. The two proteins have 20.48% sequence identity. Panels (B) and (C) ITF data showing the change in Trp fluorescence (*F* − *F*_0_/*F*_0_ where *F*_0_ is initial fluorescence in the absence of detergent) as a function of increasing concentrations of ODG or Tween 20 in the presence of 100 nM peptide/HLA (green circles) or 100 nM endo/hCD1d (purple triangles) or at 25°C. Fits of the binding isotherms are shown only when saturation was achieved. Each data is the mean ± standard deviation for three replicates. Panels (D) and (E) MST data showing the change in normalized fluorescence signal (*F*_norm_, black circles) as a function of increasing concentrations of ODG or Tween 20 in the presence of 100 nM AF647‐labeled peptide/HLA (green circles) or 100 nM AF‐647‐labeled endo/hCD1d (purple triangles) at 25°C. Fits of the binding isotherms are shown. Each data is the mean ± standard deviation for three replicates. Panels (F) and (G) NanoDSF data showing the change in melting temperature (*T*_m_) as a function of increasing concentrations of ODG or Tween 20 in the presence of 1 μM peptide/HLA (green circles) or 1 μM endo/hCD1d (purple triangles). Each data is the mean ± standard deviation for three replicates. For each detergent concentration, the *T*_m_ was determined from the inflection point of the first derivative curve of the Trp fluorescence ratio, ∂(*F*_350_/*F*_330_)/∂*T*. Δ*T*_m_ is defined as the *T*_m_ difference in the absence and presence of the maximum concentration of detergent. The peptide/HLA is the CSP 319‐328/HLA‐B*08:01/hβ2M complex described previously (Frooman et al., 2025).
**Supplementary Figure S11.** Binding of *α*‐GalCer to hCD1d by ITF, MST, and nanoDSF in the presence of Tween 20. (A) ITF data showing the change in Trp fluorescence (*F* − *F*_0_/*F*_0_, where *F*_0_ is initial fluorescence in the absence of detergent) as a function of increasing concentrations of α‐GalCer in the presence of 100 nM hCD1d with 0.05% Tween 20 (black circles) at 25°C. Fits of the binding isotherms are shown with a red line. Each data is mean ± standard deviation for three replicates. (B) NanoDSF data showing the change in the first derivative of the Trp fluorescence ratio, ∂(*F*_350_/*F*_330_)/∂*T*, as a function of increasing concentrations of *α*‐GalCer with 1 μM hCD1d with 0.05% Tween 20. The color gradient denotes the concentration range of *α*‐GalCer, where the DSF spectra are color‐coded. The dotted arrows highlight an *α*‐GalCer‐dependent increase in the melting temperature (*T*_m_) of hCD1d. Δ*T*_m_ is defined as the *T*_m_ difference between CD1d in the absence and presence of 50 μM *α*‐GalCer. Each data is the mean ± standard deviation for three replicates. (C) MST data showing the change in normalized fluorescence signal (*F*_norm_, black circles) as a function of increasing concentrations of *α*‐GalCer with 100 nM AF647‐labeled hCD1d with 0.05% Tween 20 at 25°C. Each data is the mean ± standard deviation for three replicates.
**Supplementary Figure S12.** Comparison of predicted detergent binding residues across human CD1 isoforms. Protein sequence alignment for the ectodomain of human CD1 molecules with Clustal Omega v1.2.4 and visualized in ESPript v3.0. Sequences used: hCD1a (UniProt #P06126), hCD1b (UniProt #P29016), hCD1c (UniProt #P29017), and hCD1d (UniProt #P15813). The blue triangles represent hCD1d residues predicted to interact with detergents based on Chai‐1 models (see Table S2).
**Table S1.** Chai‐1 model confidence metrics for the detergent/CD1d ortholog complex models.
**Table S2.** Conserved interaction residues in Chai‐1 models of detergent/hCD1d complexes relative to *α*‐GalCer/hCD1d.

**Data S1:** Supplementary Information

## Data Availability

All raw source data, scripts, and computational models are freely provided on GitHub at https://github.com/mcshanlab/Miles_et-al_CD1d_detergent.
