# Supplementary material for: Detergents alter the stability and lipid binding properties of the CD1d immunoreceptor
Source: Protein Sci. 2025 Dec 22;35(1):e70417. doi: 10.1002/pro.70417 (PMC12720774; doi:10.1002/pro.70417)
Supplement: Supplementary file 1 — Supplementary Figure S1. Purification and characterization of a recombinant single‐chain construct of hCD1d. (A) Following a Ni‐NTA affinity column, the second round purification of size exclusion chromatography purification of hCD1d with a Superdex 200 Increase 10/300 GL column at 0.5 mL/min in 1X PBS pH 7.4. (B) SDS‐PAGE of purified hCD1d with PageRuler Unstained Protein Ladder (Thermo Fisher Scientific #26614). The protein travels higher than its expected molecular weight (~47.5 kDa) due to glycosylation from mammalian cell expression. (C) Far‐UV circular dichroism spectra of 0.35 mg/mL hCD1d recorded at 25°C recorded in the absence of detergent. The characteristic negative band near 218 nm corresponds to β‐sheet secondary structure, which is the main component of the immunoglobulin fold of CD1d and β2m. The CD spectra profile is also consistent with previously acquired CD spectra of CD1 molecules. (D) First derivative of the nanoDSF spectra (F 350/F 330) of 1 μM hCD1d recorded in the absence of detergent. The fitted melting temperature (T m) is noted. Supplementary Figure S2. Raw ITF data. Fluorescence spectra of tryptophan from 100 nM hCD1d in the absence and presence of increasing amounts of detergents acquired at 25°C. Supplementary Figure S3. Raw MST data. Raw MST traces of 100 nM AF647‐hCD1d in the absence and presence of increasing amounts of detergents acquired at medium MST power at 25°C with LED power set between 30% and 50%. Supplementary Figure S4. Determination of ΔT m values from nanoDSF data. Each plot shows the nanoDSF determined melting temperature (T m) of hCD1d as a function of increasing concentrations of detergents (μM). For each detergent concentration, the schCD1d T m was determined from the inflection point of the first derivative curve of the Trp fluorescence ratio, ∂(F 350/F 330)/∂T. The red arrows highlight the detergent concentration at which the ∂(F 350/F 330)/∂T goes into the noise. Each data point is the mean ± standard deviation fo [file PRO-35-e70417-s002.docx]

**Supplementary Information**


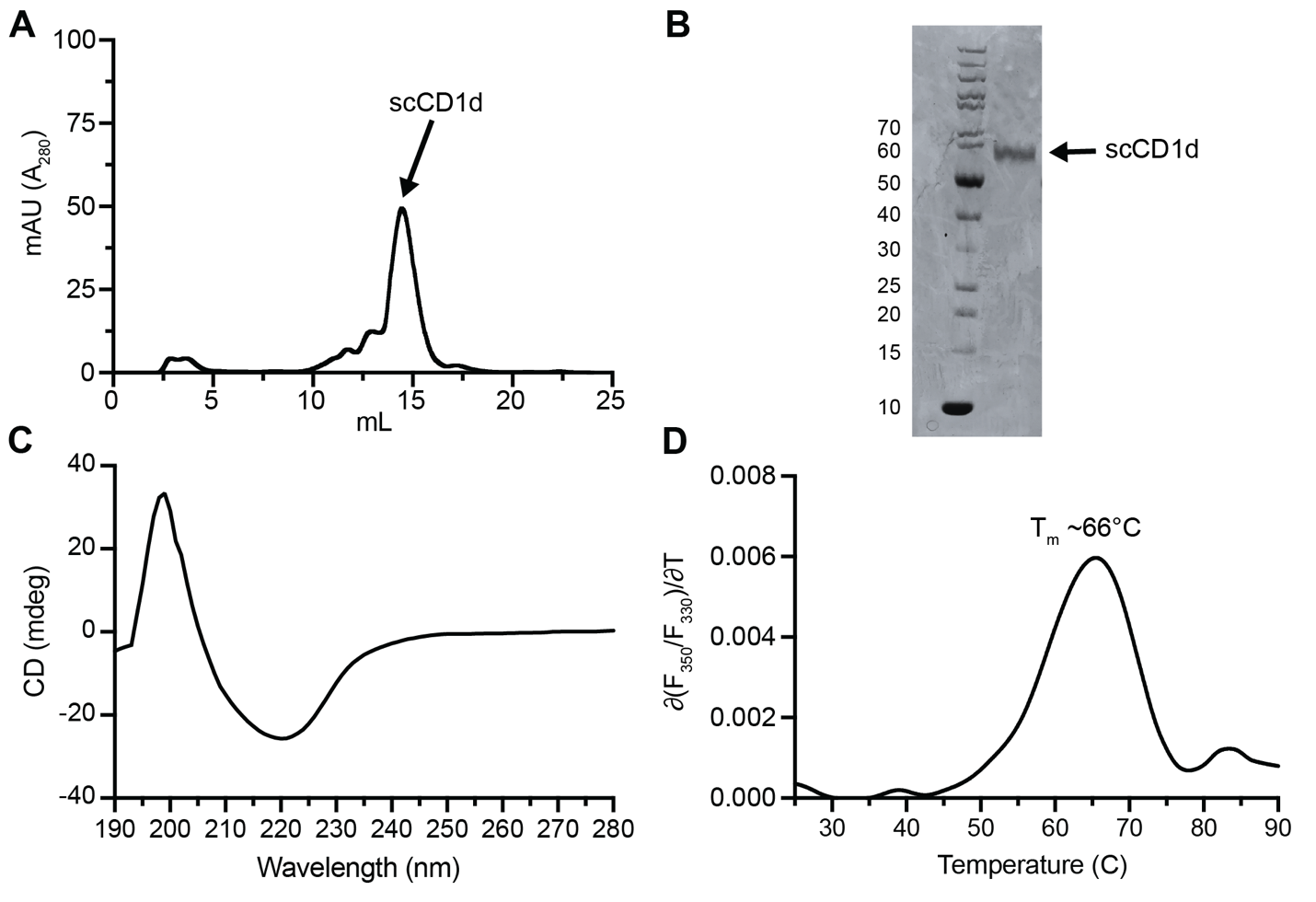


**Supplementary Figure S1. Purification and characterization of a recombinant single-chain construct of hCD1d.** (A) Following a Ni-NTA affinity column, the second round purification of size exclusion chromatography purification of hCD1d with a Superdex 200 Increase 10/300 GL column at 0.5 mL/min in 1X PBS pH 7.4. (B) SDS-PAGE of purified hCD1d with PageRuler Unstained Protein Ladder (Thermo Fisher Scientific #26614). The protein travels higher than its expected molecular weight (~47.5 kDa) due to glycosylation from mammalian cell expression. (C) Far-UV circular dichroism spectra of 0.35 mg/mL hCD1d recorded at 25°C recorded in the absence of detergent. The characteristic negative band near 218 nm corresponds to β-sheet secondary structure, which is the main component of the immunoglobulin fold of CD1d and β2m. The CD spectra profile is also consistent with previously acquired CD spectra of CD1 molecules (66, 67). (D) First derivative of the nanoDSF spectra (F_350_/F_330_) of 1 µM hCD1d recorded in the absence of detergent. The fitted melting temperature (T_m_) is noted.


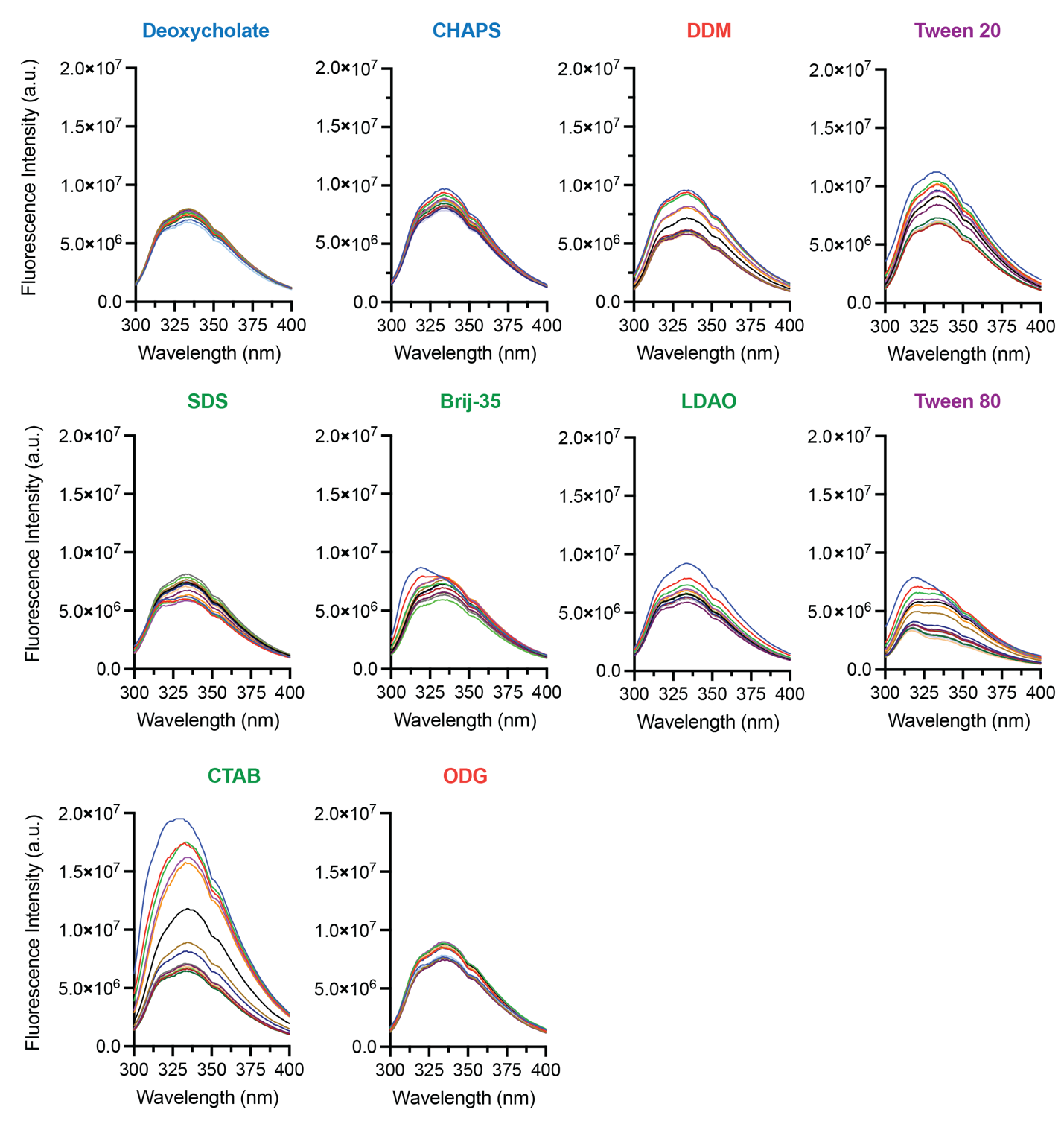


**Supplementary Figure S2. Raw ITF data.** Fluorescence spectra of tryptophan from 100 nM hCD1d in the absence and presence of increasing amounts of detergents acquired at 25°C.


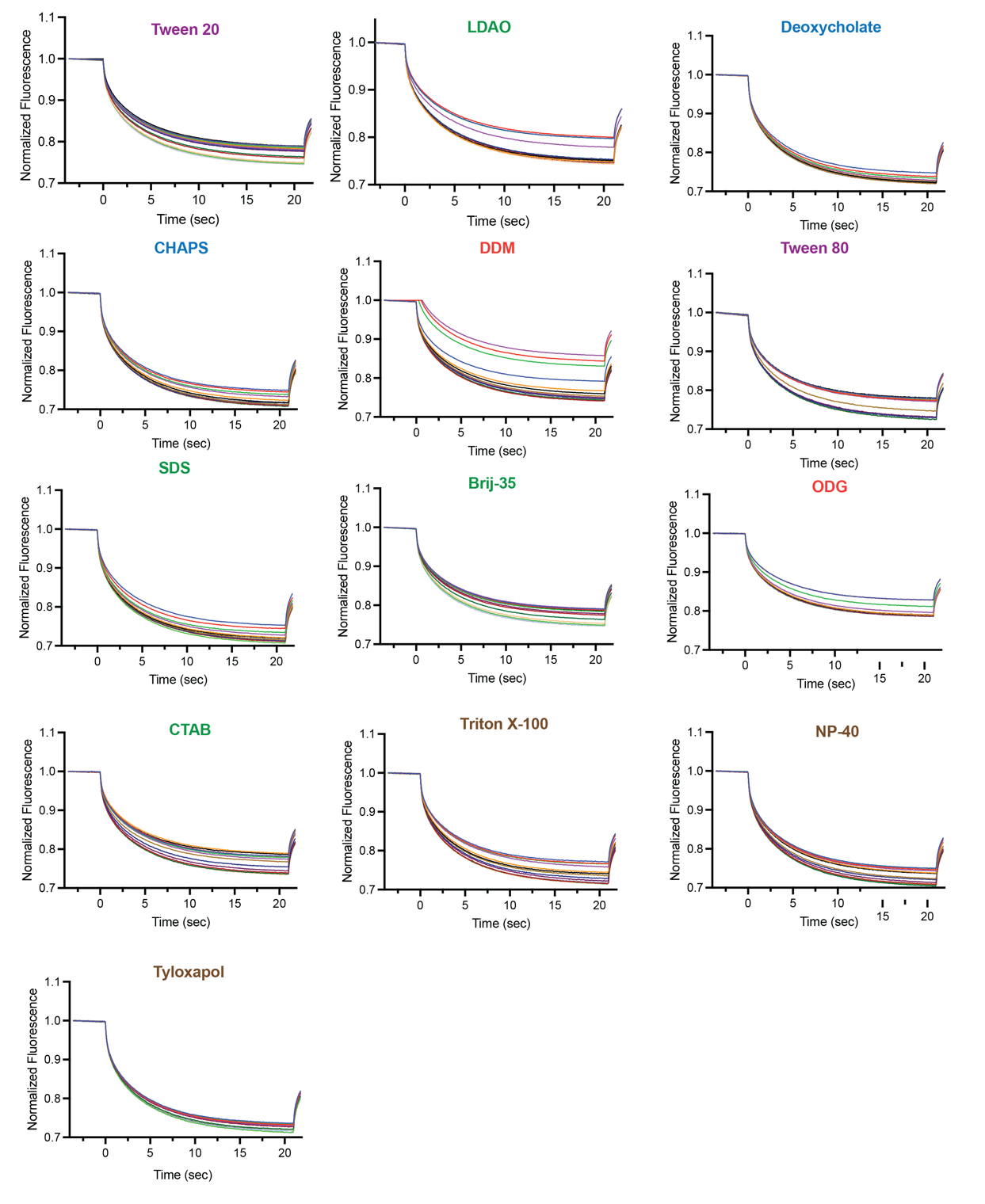


**Supplementary Figure S3. Raw MST data.** Raw MST traces of 100 nM AF647-hCD1d in the absence and presence of increasing amounts of detergents acquired at medium MST power at 25°C with LED power set between 30 and 50%.


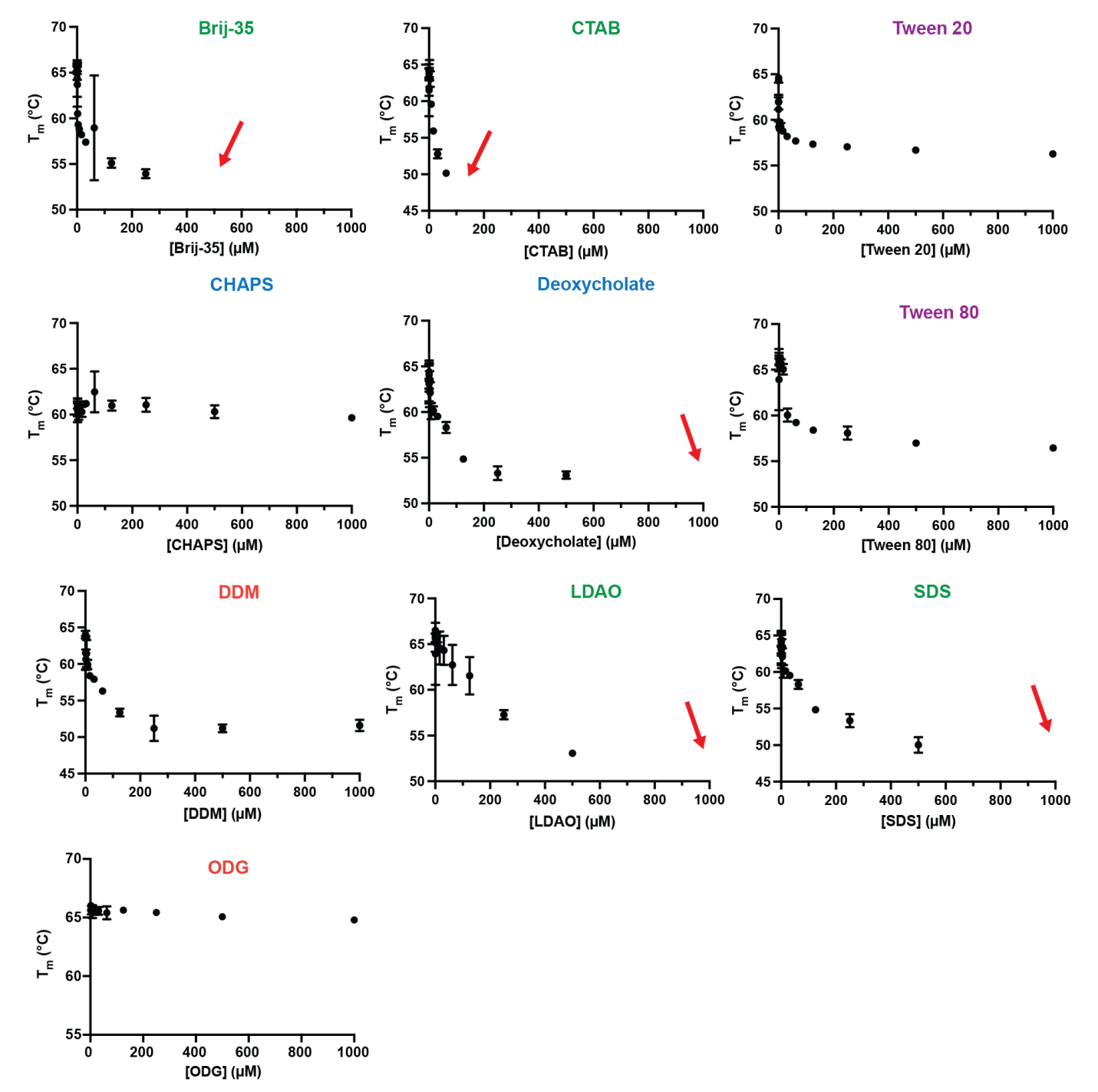


**Supplementary Figure S4. Determination of ΔT_m_ values from nanoDSF data.** Each plot shows the nanoDSF determined melting temperature (T_m_) of hCD1d as a function of increasing concentrations of detergents (µM). For each detergent concentration, the schCD1d T_m_ was determined from the inflection point of the first derivative curve of the Trp fluorescence ratio, ∂(F_350_/F_330_)/∂T. The red arrows highlight the detergent concentration upon which the ∂(F_350_/F_330_)/∂T goes into the noise. Each data point is mean ± standard deviate for three replicates.

**
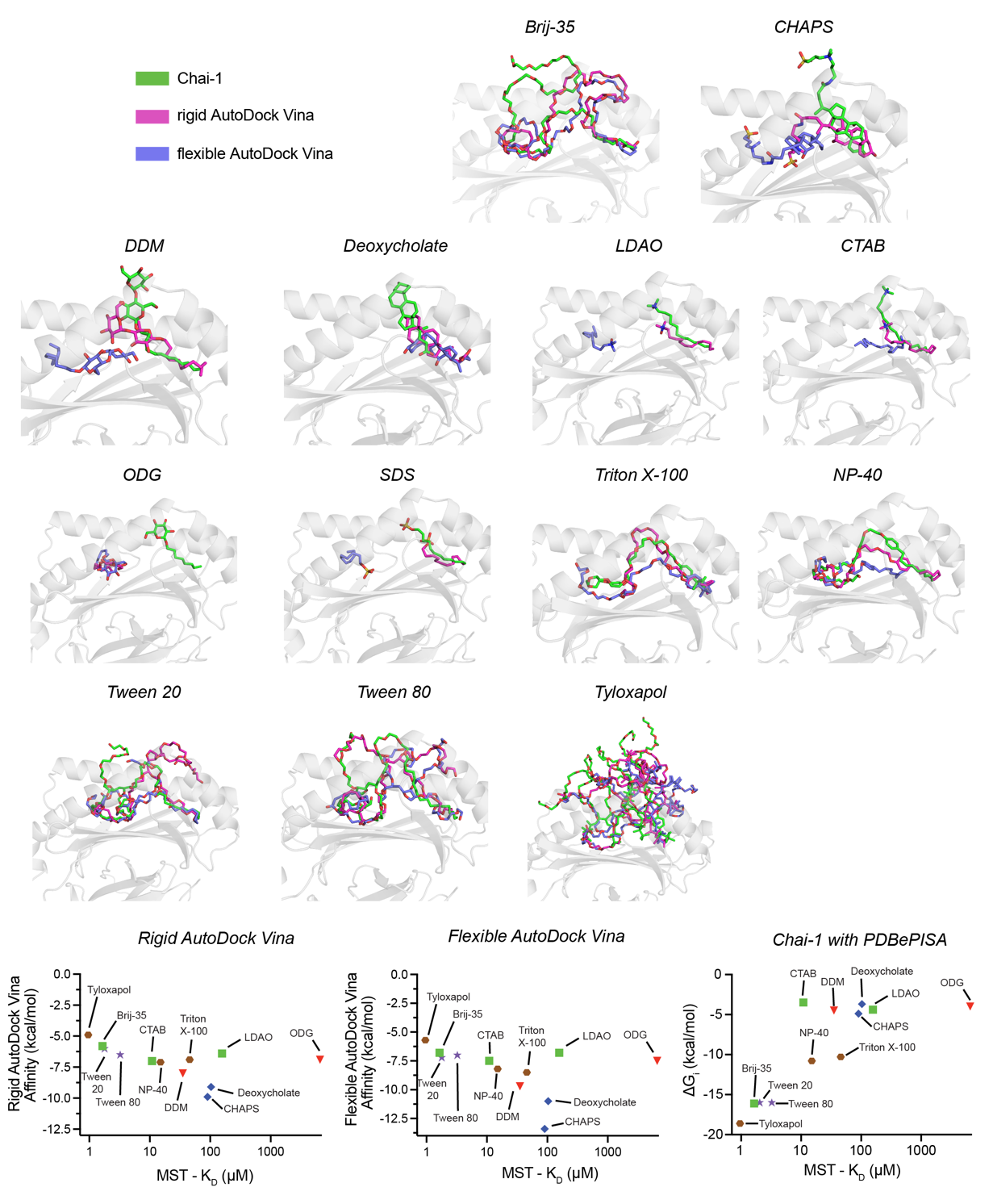
**

**Supplementary Figure S5** Modeling detergent binding poses in the hCD1d antigen binding groove with AutoDock Vina. Top: Overlay of detergent binding poses obtained from Chai-1 (green sticks), rigid AutoDock Vina (magenta sticks), and flexible AutoDock Vina (marine sticks). Bottom: AutoDock Vina predicted detergent affinity (kcal/mol) from AutoDock Vina binding poses versus MST determined K_D_ values compared with PDBePISA determined interface ΔG from Chai-1 binding poses versus MST determined K_D_ values.

**
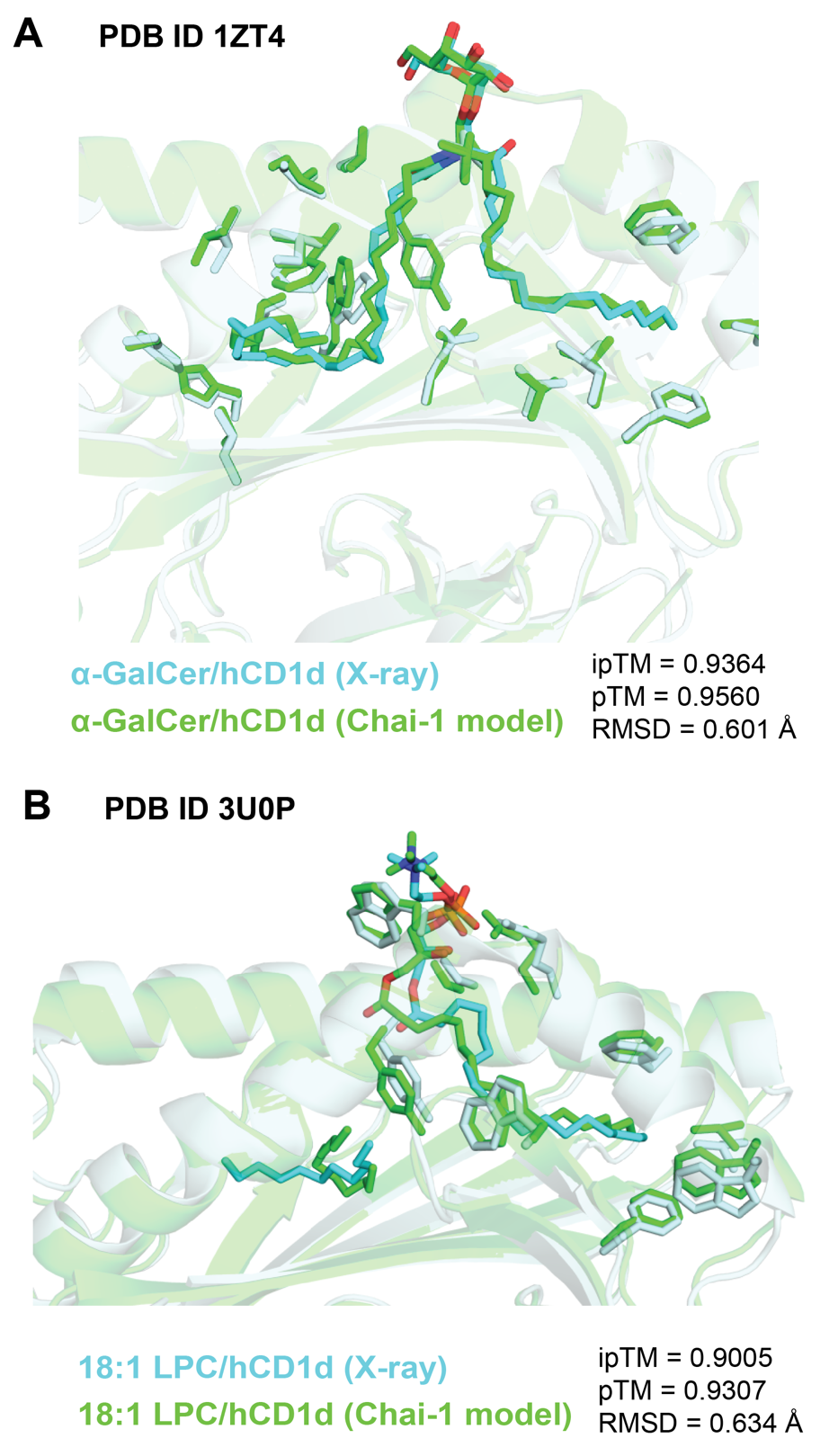
**

**Supplementary Figure S6. Comparison of *in silico* Chai-1 models with X-ray structures for native lipid antigens with hCD1d.** Overlay of Chai-1 models (green) versus X-ray structures (cyan) for α-GalCer/hCD1d (PDB ID 1ZT4) and 18:1 LPC/hCD1d (PDB ID 3U0P). Lipid atoms and CD1d residues that interact with the lipid antigens are shown as sticks. The CD1d backbone is shown as a cartoon. Chai-1 model confidence (ipTM, pTM) and heavy chain RMSD values (determined by PyMOL v3.1.6.1) are noted.


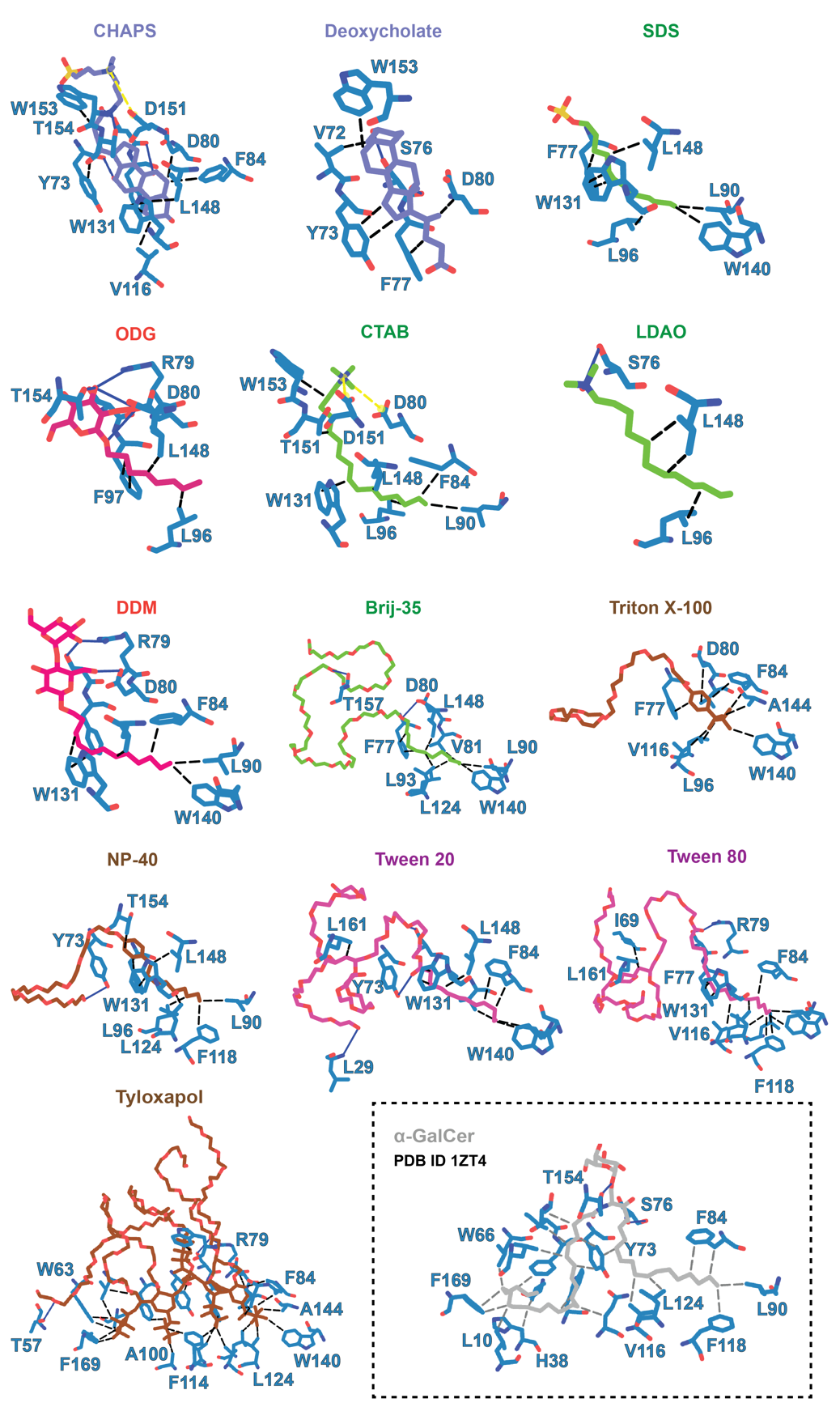


**Supplementary Figure S7. Details of detergent/hCD1d interactions from *in silico* Chai-1 models.** Molecular interactions between detergents and hCD1d groove from the Chai-1 models. Hydrogen bonds (blue dashes), hydrophobic interactions (black dashes), and salt bridges (yellow dashes) are predicted by the PLIP tool (63). Models are oriented as shown in Figure 6. CD1d residues are shown as dark blue sticks; the CD1d backbone cartoon is not shown for clarity.

**
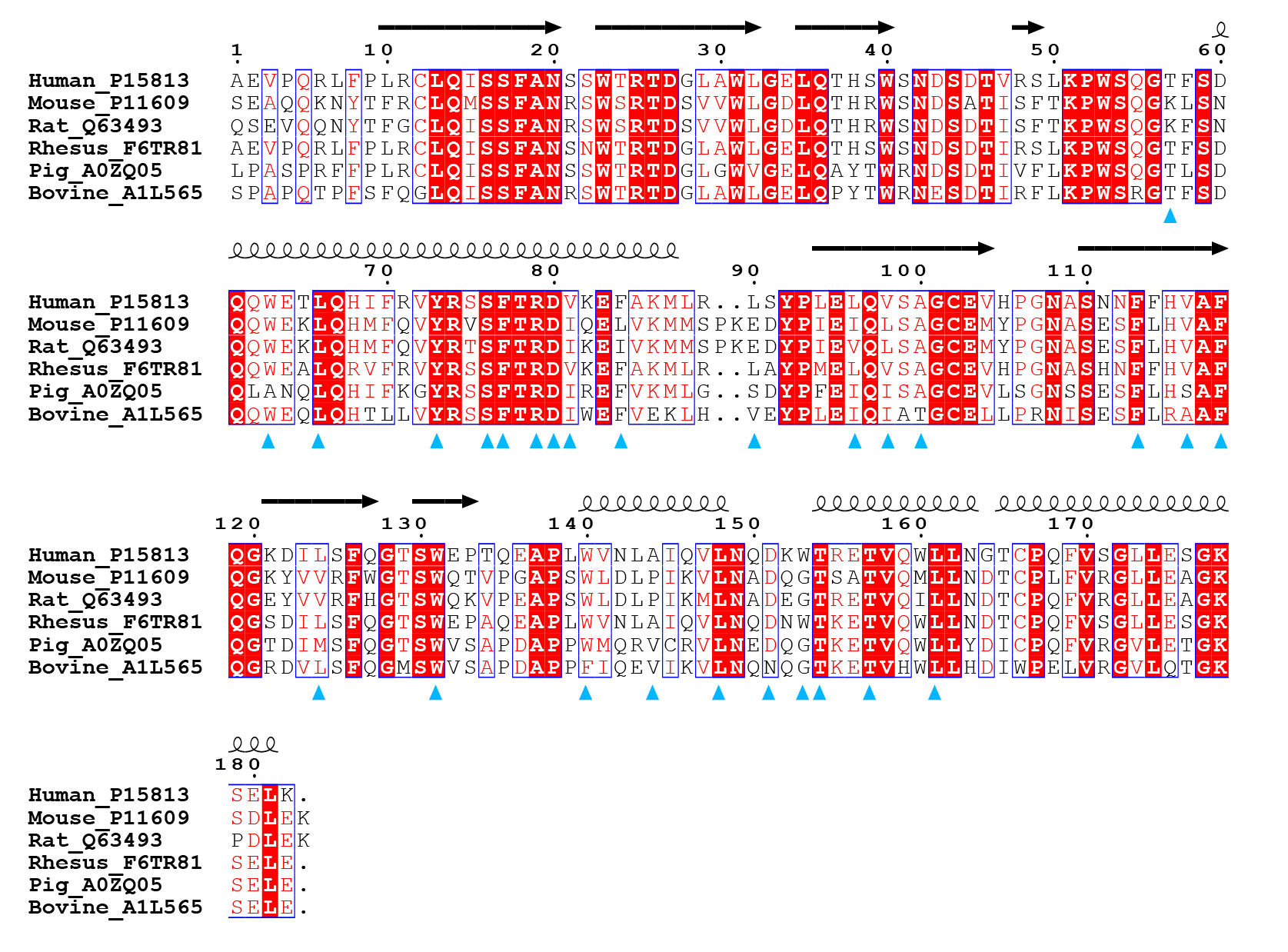
**

**Supplementary Figure S8**. Comparison of predicted detergent binding residues across the antigen binding groove residues of CD1d orthologs. Protein sequence alignment was performed for the ectodomain of CD1d molecules with Clustal Omega v1.2.4 and visualized in ESPript v3.0. Sequences used: *Homo sapiens* CD1d (human, UniProt #P15813), *Mus musculus* (mouse, UniProt #P11609), *Rattus norvegicus* (rat, UniProt #Q63493), *Macaca mulatta* (rhesus macaque, UniProt #F6TR81), *Sus scrofa* (pig, UniProt #A0ZQ05), and *Bos taurus* (bovine, UniProt #A1L565). The blue triangles represent CD1d residues predicted to interact with detergents based on Chai-1 models (see Table S2).

**
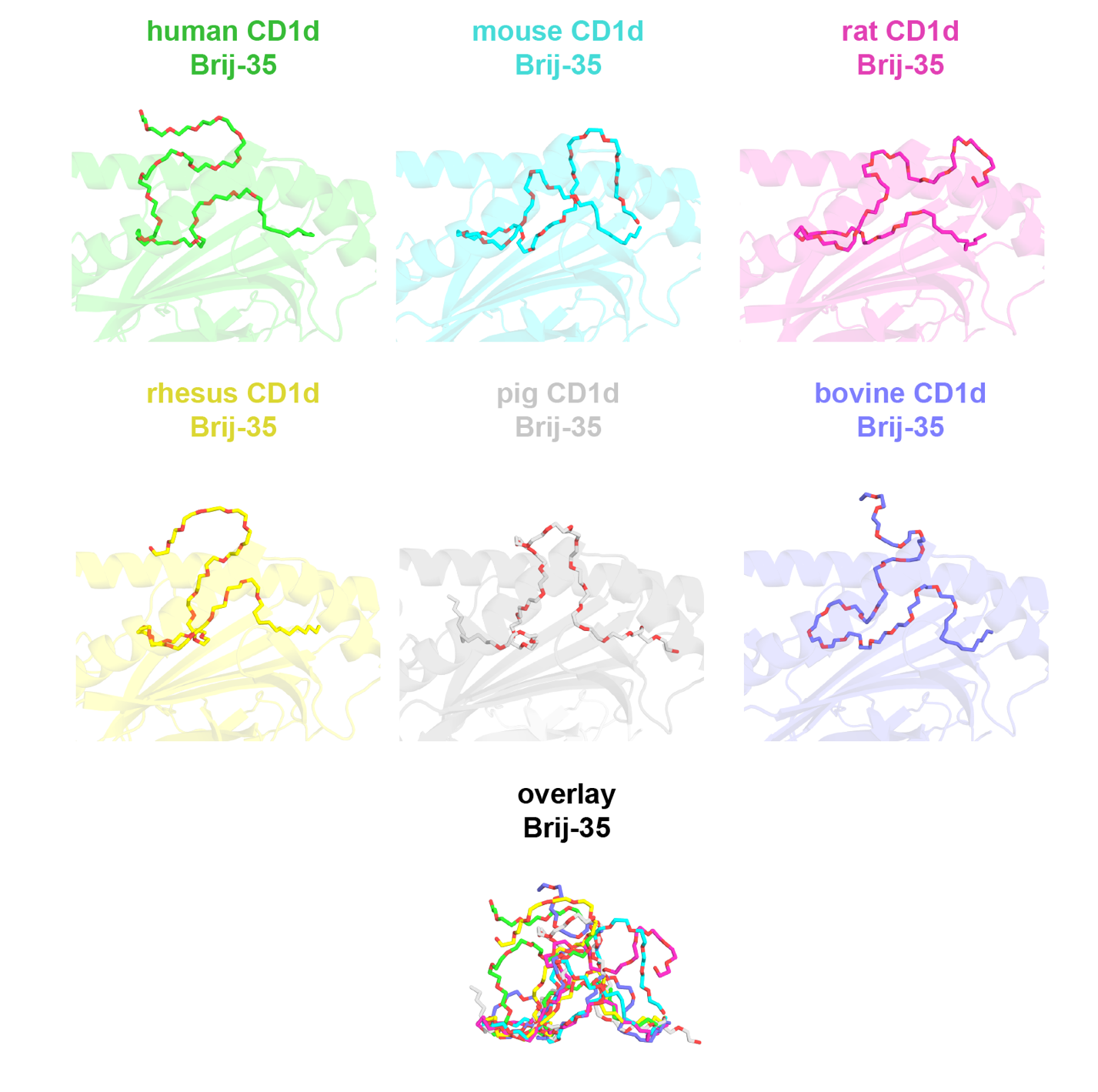
**

**Supplementary Figure S9**. Chai-1 modes of CD1d orthologs with different detergents.

**
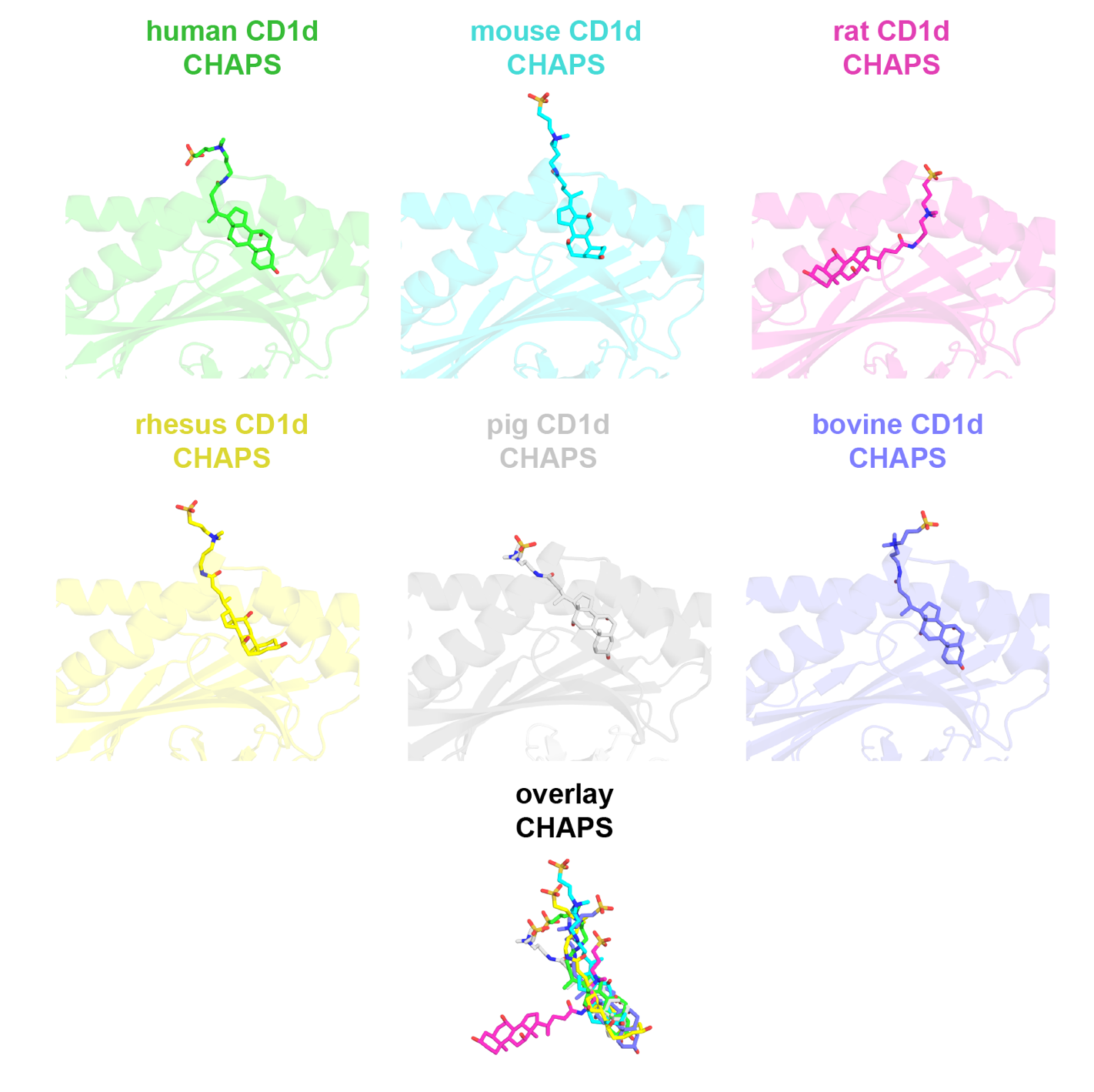
**

**Supplementary Figure S9 cont**. Chai-1 modes of CD1d orthologs with different detergents.

**
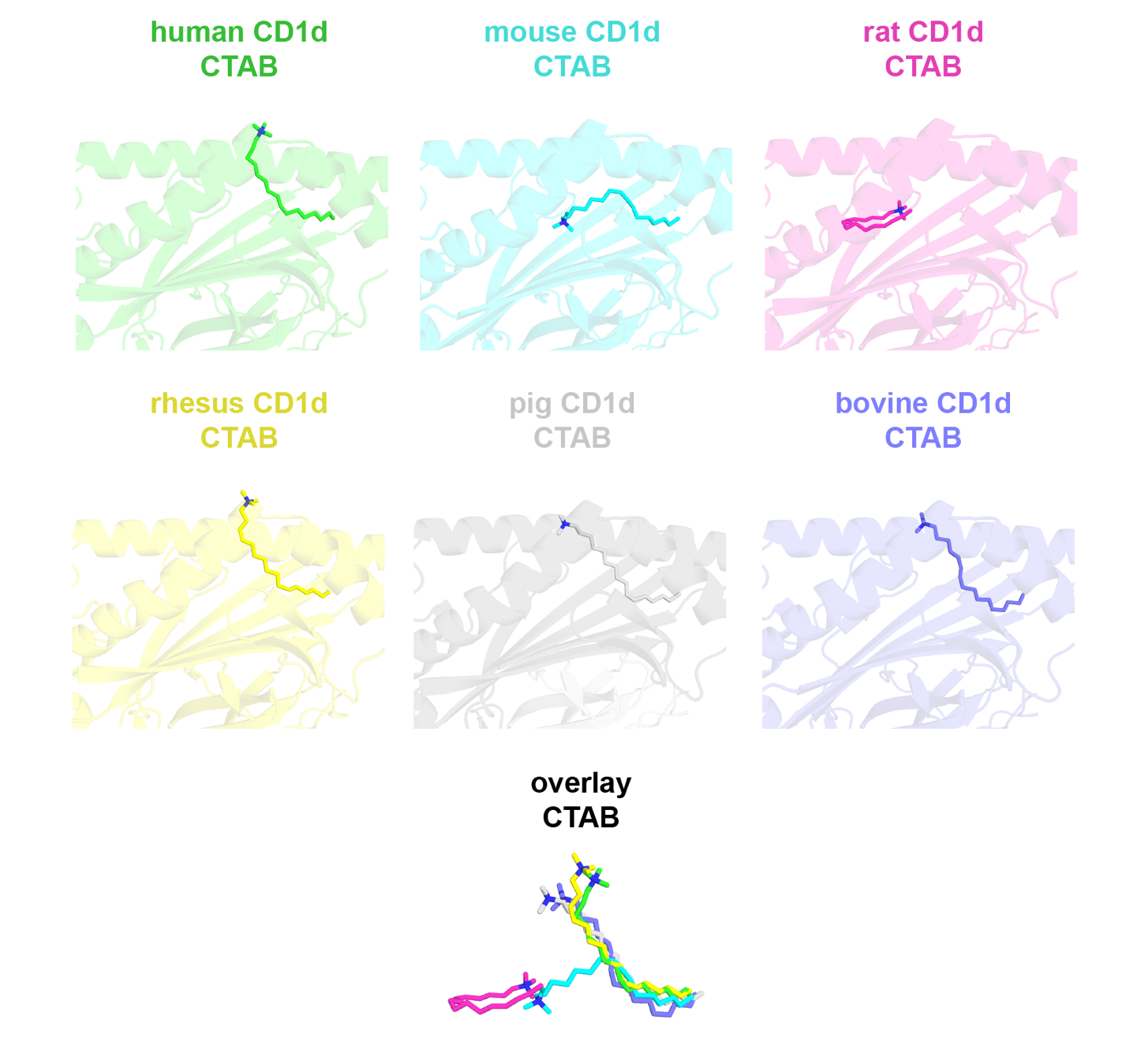
**

**Supplementary Figure S9 cont**. Chai-1 modes of CD1d orthologs with different detergents.

**
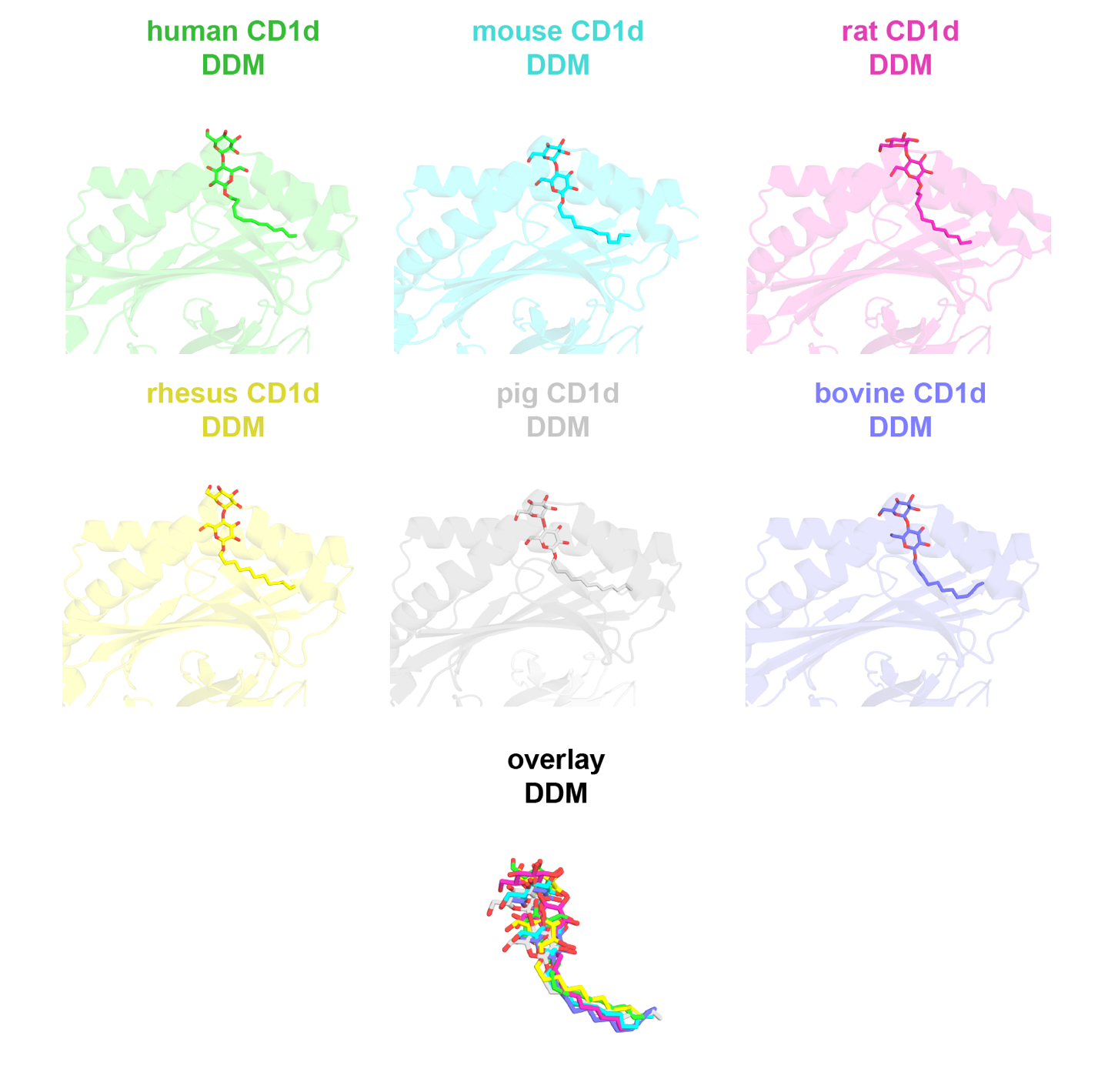
**

**Supplementary Figure S9 cont**. Chai-1 modes of CD1d orthologs with different detergents.

**
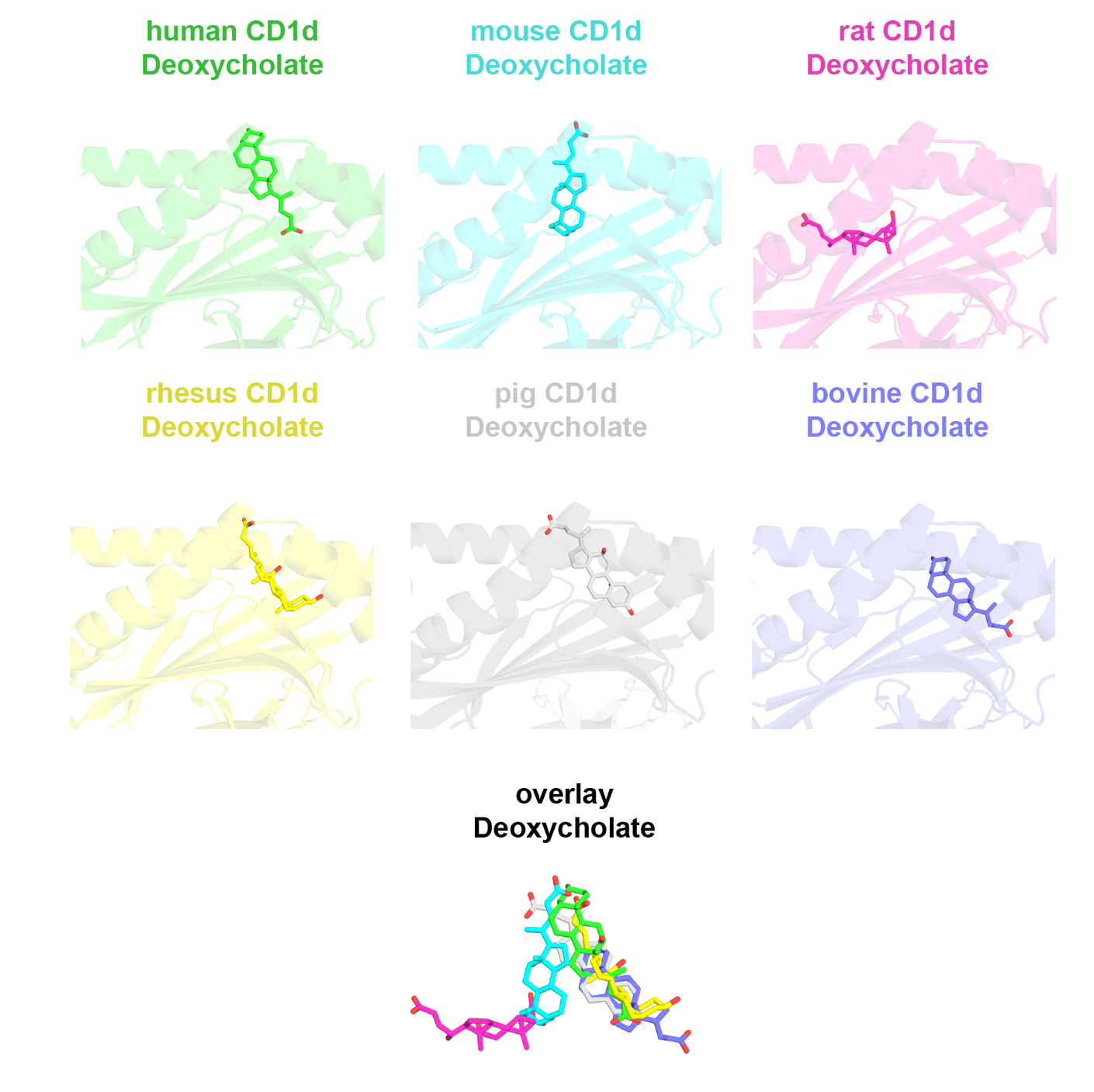
**

**Supplementary Figure S9 cont**. Chai-1 modes of CD1d orthologs with different detergents.

**
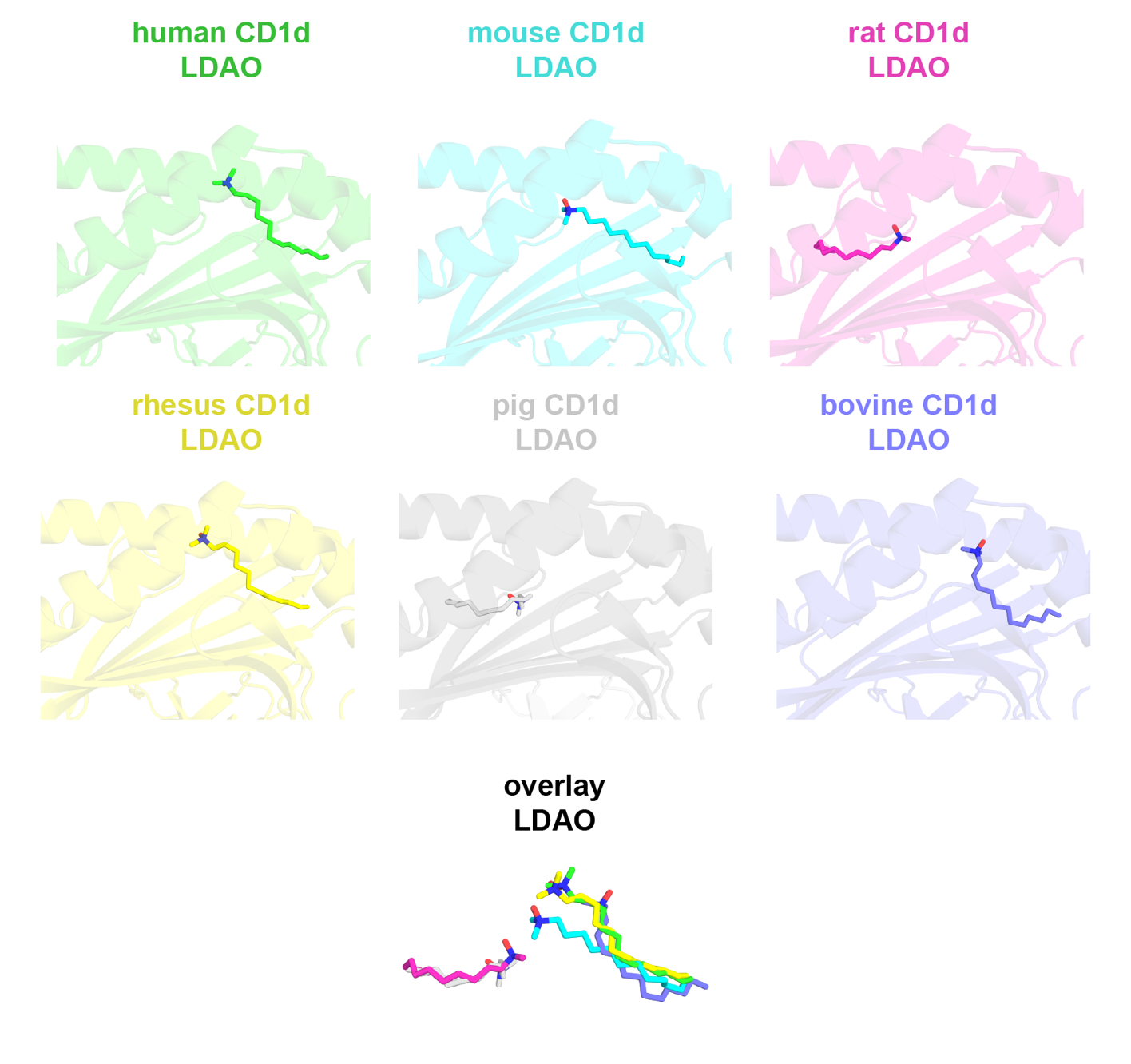
**

**Supplementary Figure S9 cont**. Chai-1 modes of CD1d orthologs with different detergents.

**
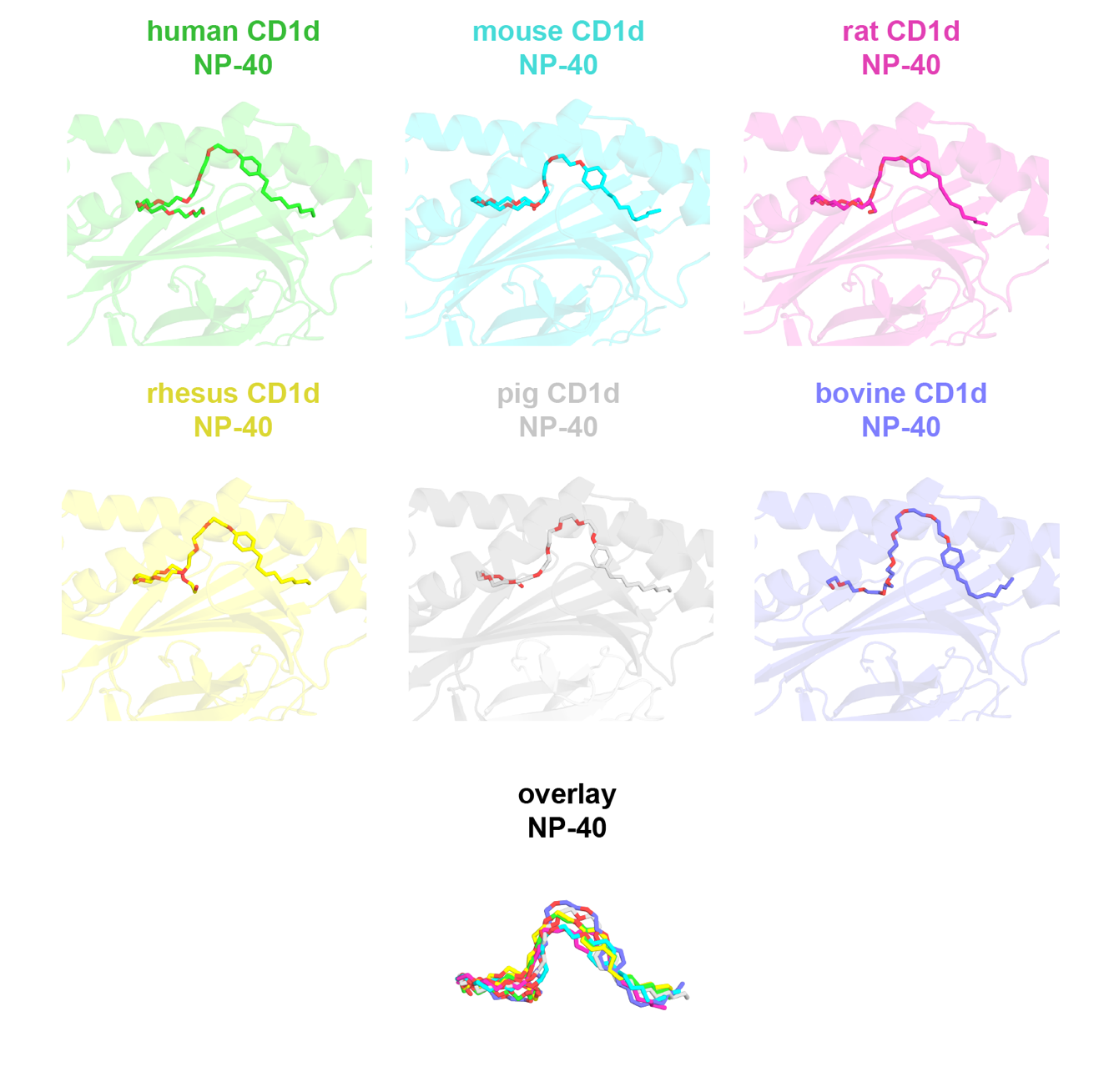
**

**Supplementary Figure S9 cont**. Chai-1 modes of CD1d orthologs with different detergents.

**
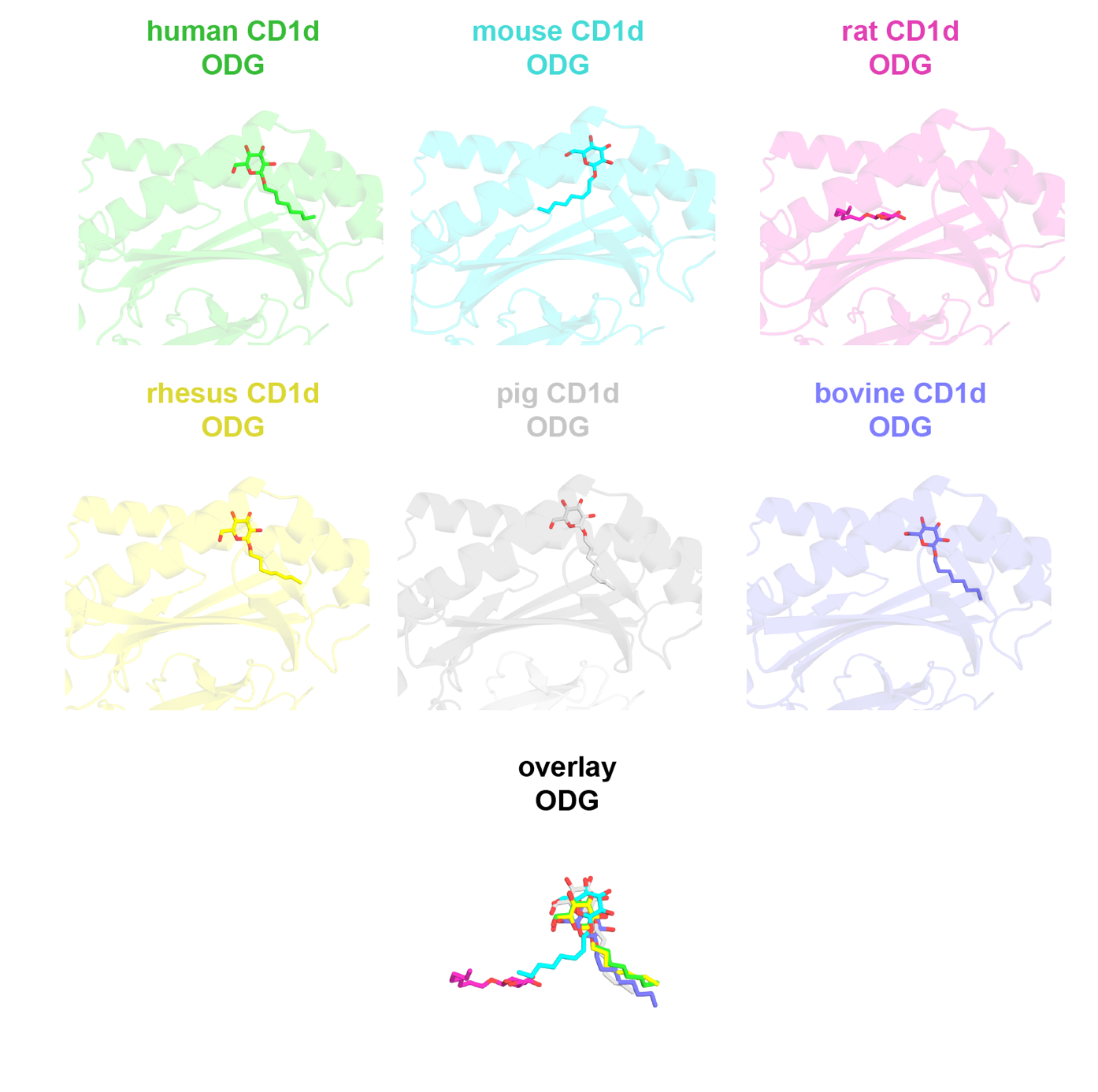
**

**Supplementary Figure S9 cont**. Chai-1 modes of CD1d orthologs with different detergents.

**
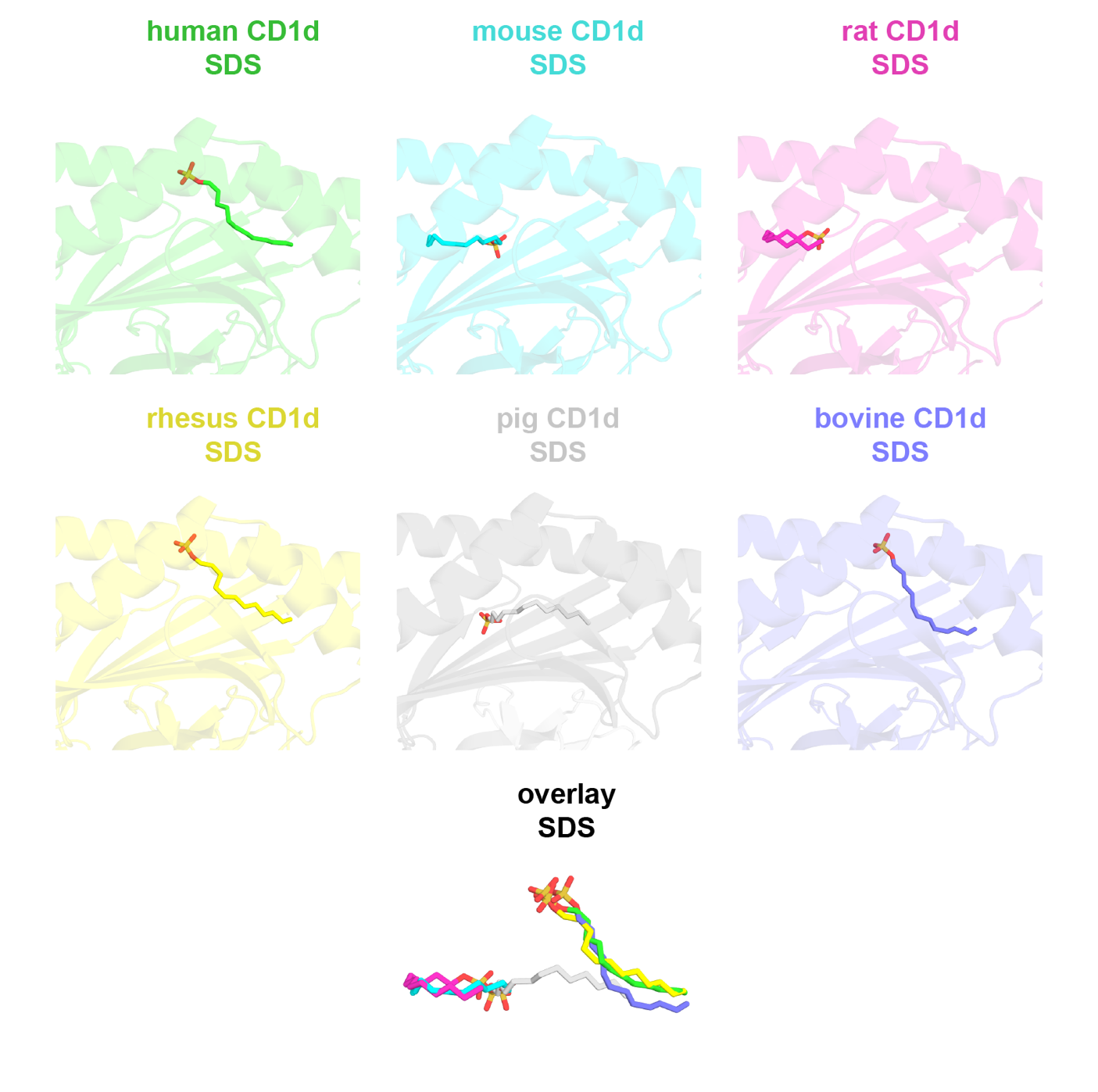
**

**Supplementary Figure S9 cont**. Chai-1 modes of CD1d orthologs with different detergents.

**
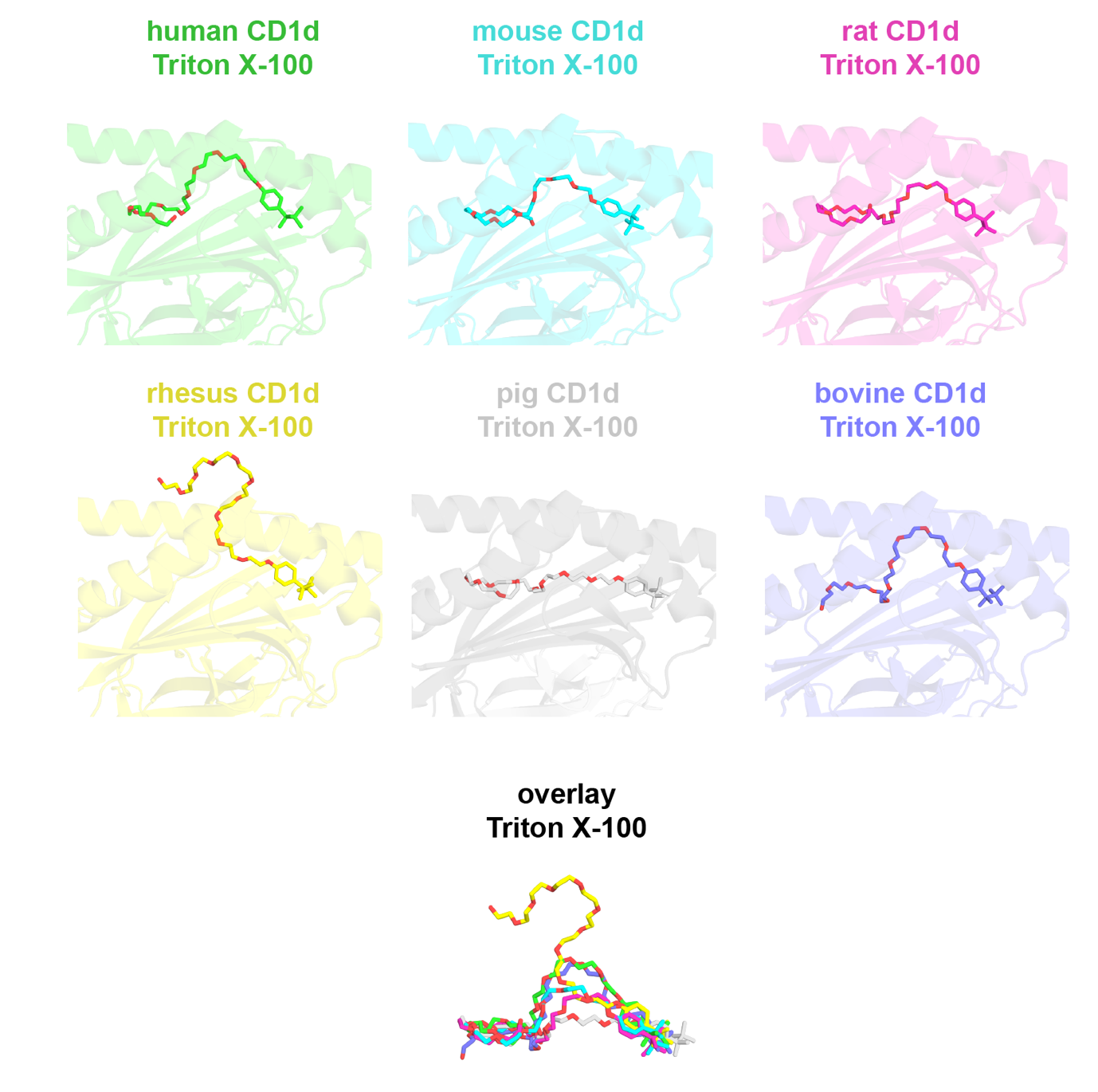
**

**Supplementary Figure S9 cont**. Chai-1 modes of CD1d orthologs with different detergents.

**
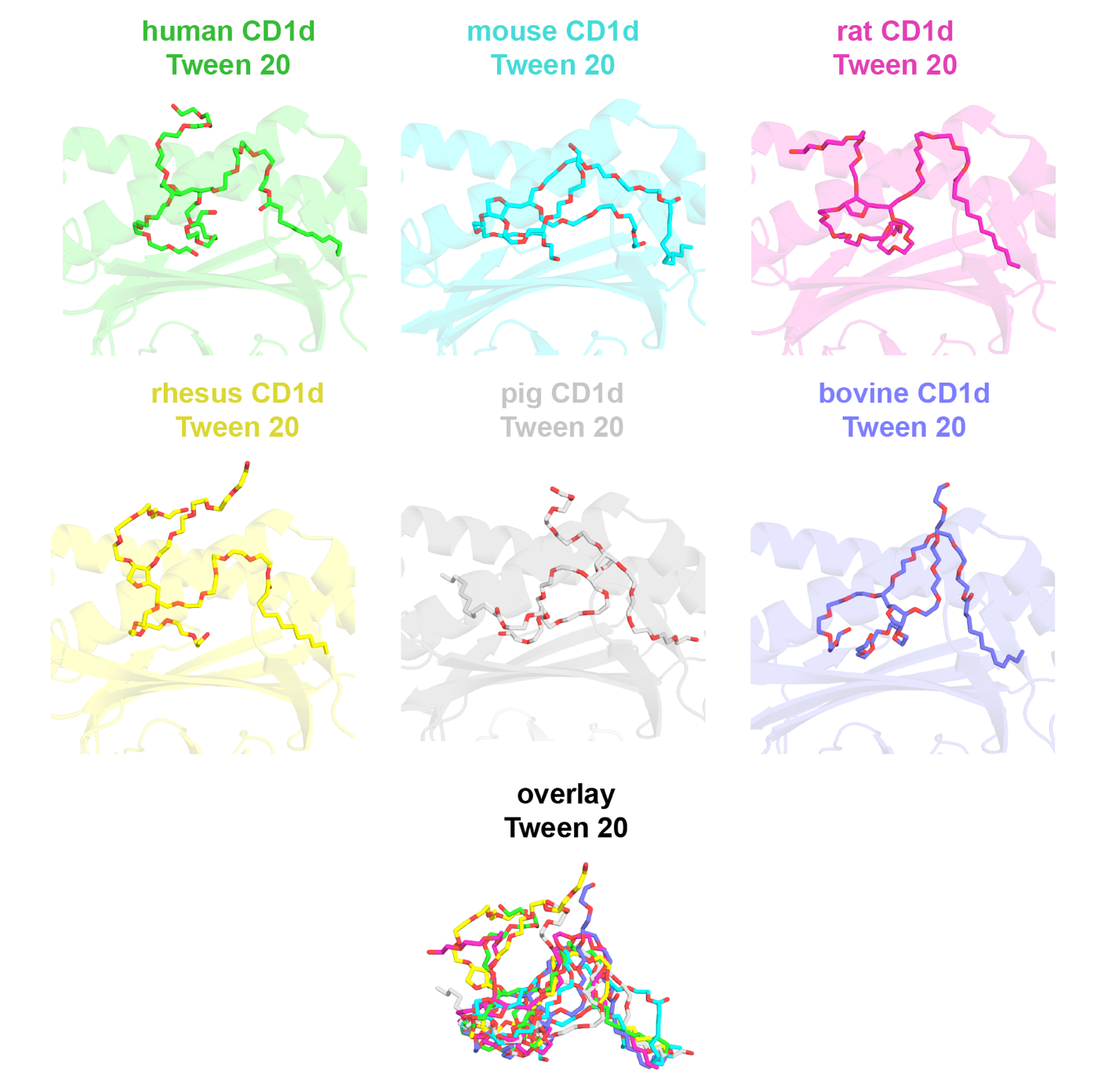
**

**Supplementary Figure S9 cont**. Chai-1 modes of CD1d orthologs with different detergents.

**
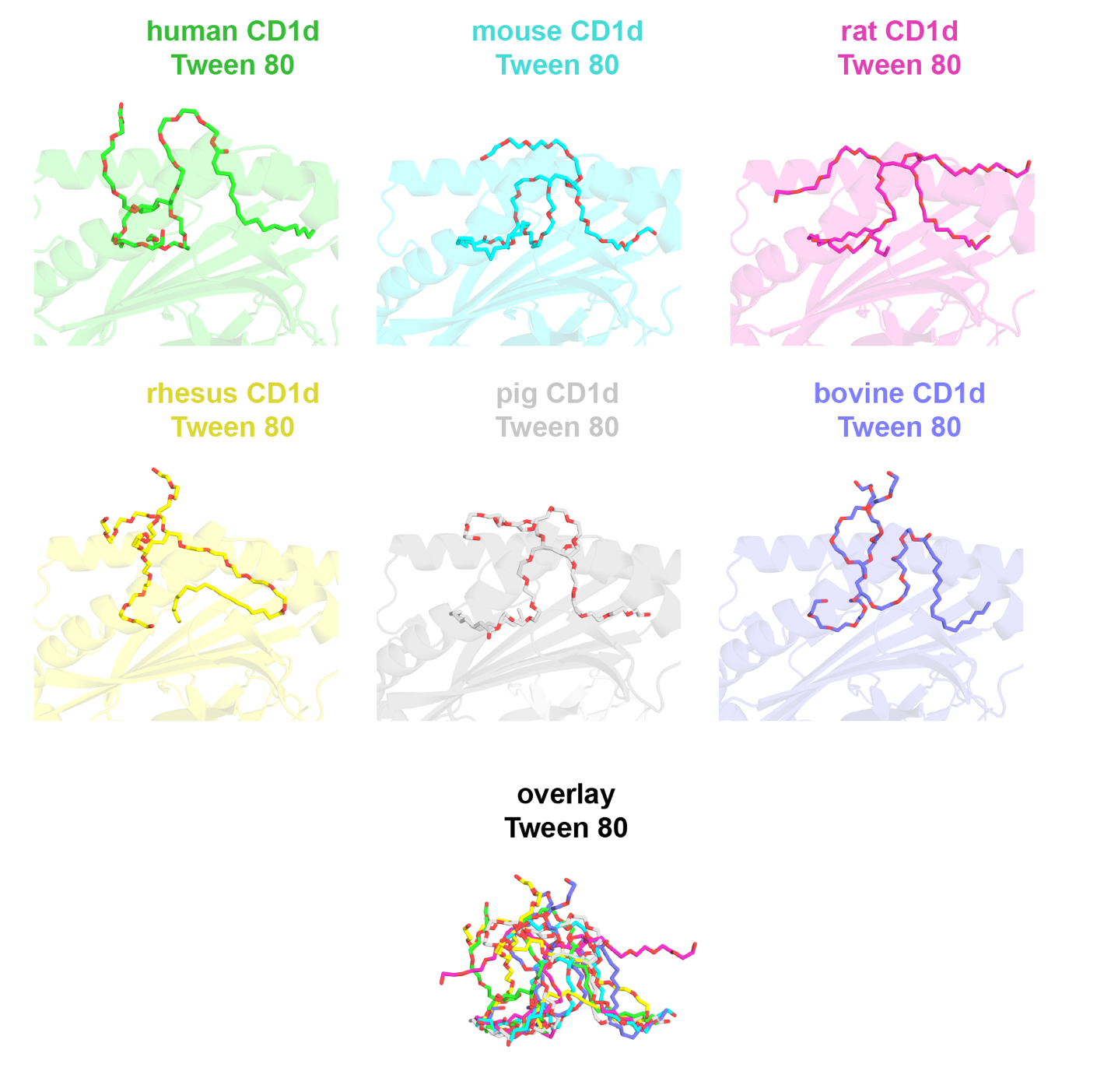
**

**Supplementary Figure S9 cont**. Chai-1 modes of CD1d orthologs with different detergents.

**
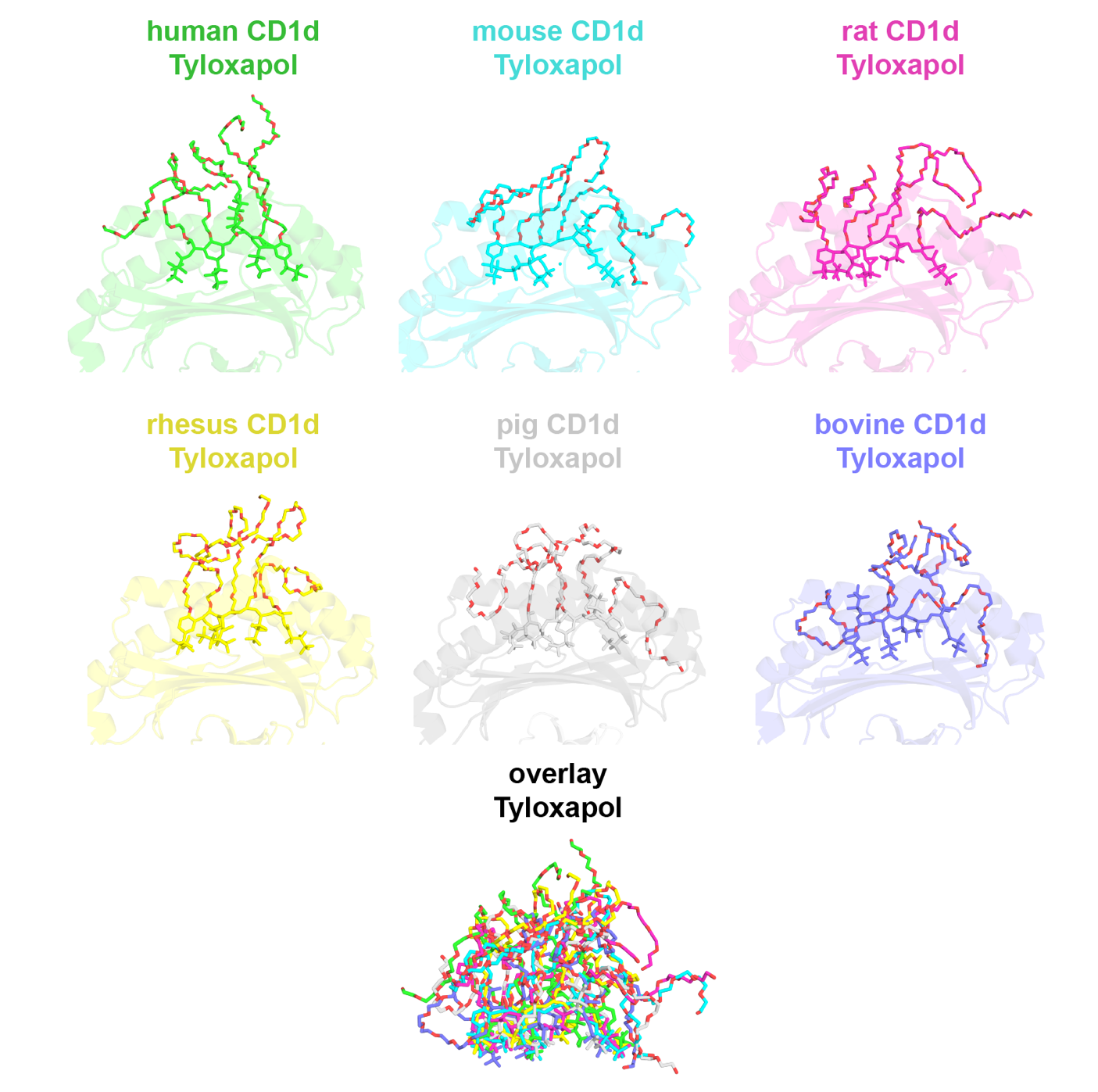
**

**Supplementary Figure S9 cont**. Chai-1 modes of CD1d orthologs with different detergents.

**
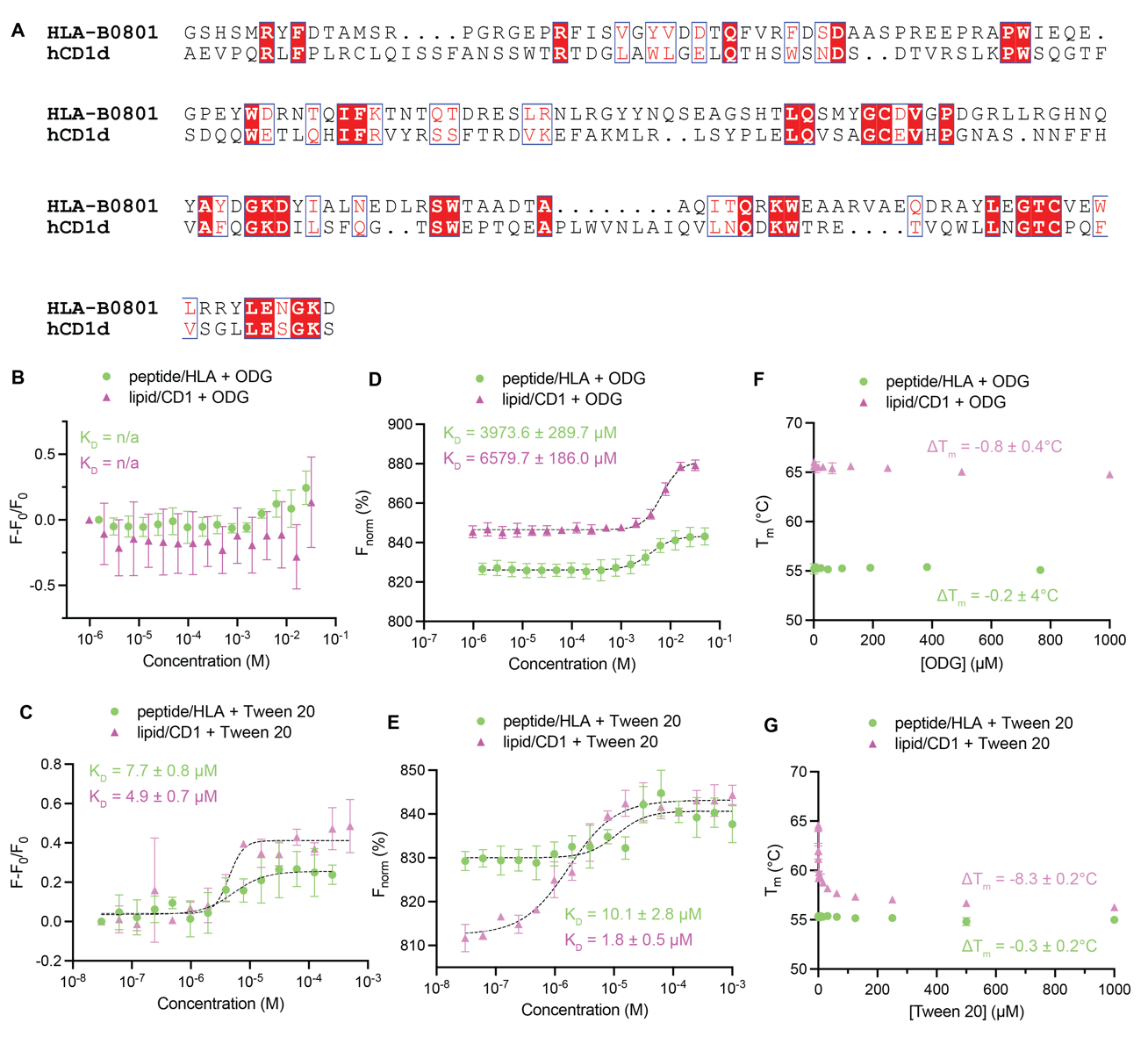
**

**Supplementary Figure S10. Binding of ODG and Tween 20 to a peptide/HLA complex by ITF, MST, and nanoDSF.** (A) Sequence alignment for the antigen binding groove residues of the ectodomain of human HLA-B*08:01 (UniProt #P01889) and human CD1d (UniProt #P15813) with Clustal Omega v1.2.4 and visualized in ESPript v3.0. The two proteins have 20.48% sequence identity. Panels (B) and (C) ITF data showing the change in Trp fluorescence (F-F_0_/F_0_ where F_0_ is initial fluorescence in the absence of detergent) as a function of increasing concentrations of ODG or Tween 20 in the presence of 100 nM peptide/HLA (green circles) or 100 nM endo/hCD1d (purple triangles) or at 25°C. Fits of the binding isotherms are as shown only when saturation was achieved. Each data is mean ± standard deviation for three replicates. Panels (D) and (E) MST data showing the change in normalized fluorescence signal (F_norm_, black circles) as a function of increasing concentrations of ODG or Tween 20 in the presence of 100 nM AF647-labeled peptide/HLA (green circles) or 100 nM AF-647-labeled endo/hCD1d (purple triangles) at 25°C. Fits of the binding isotherms are shown. Each data is mean ± standard deviation for three replicates. Panels (F) and (G) NanoDSF data showing the change in melting temperature (T_m_) as a function of increasing concentrations of ODG or Tween 20 in the presence of 1 µM peptide/HLA (green circles) or 1 µM endo/hCD1d (purple triangles). Each data is mean ± standard deviation for three replicates. For each detergent concentration, the T_m_ was determined from the inflection point of the first derivative curve of the Trp fluorescence ratio, ∂(F_350_/F_330_)/∂T. ΔT_m_ is defined as the T_m_ difference in the absence and presence of the maximum concentration of detergent. The peptide/HLA is the CSP 319-328/HLA-B*08:01/hβ2M complex described previously (57).


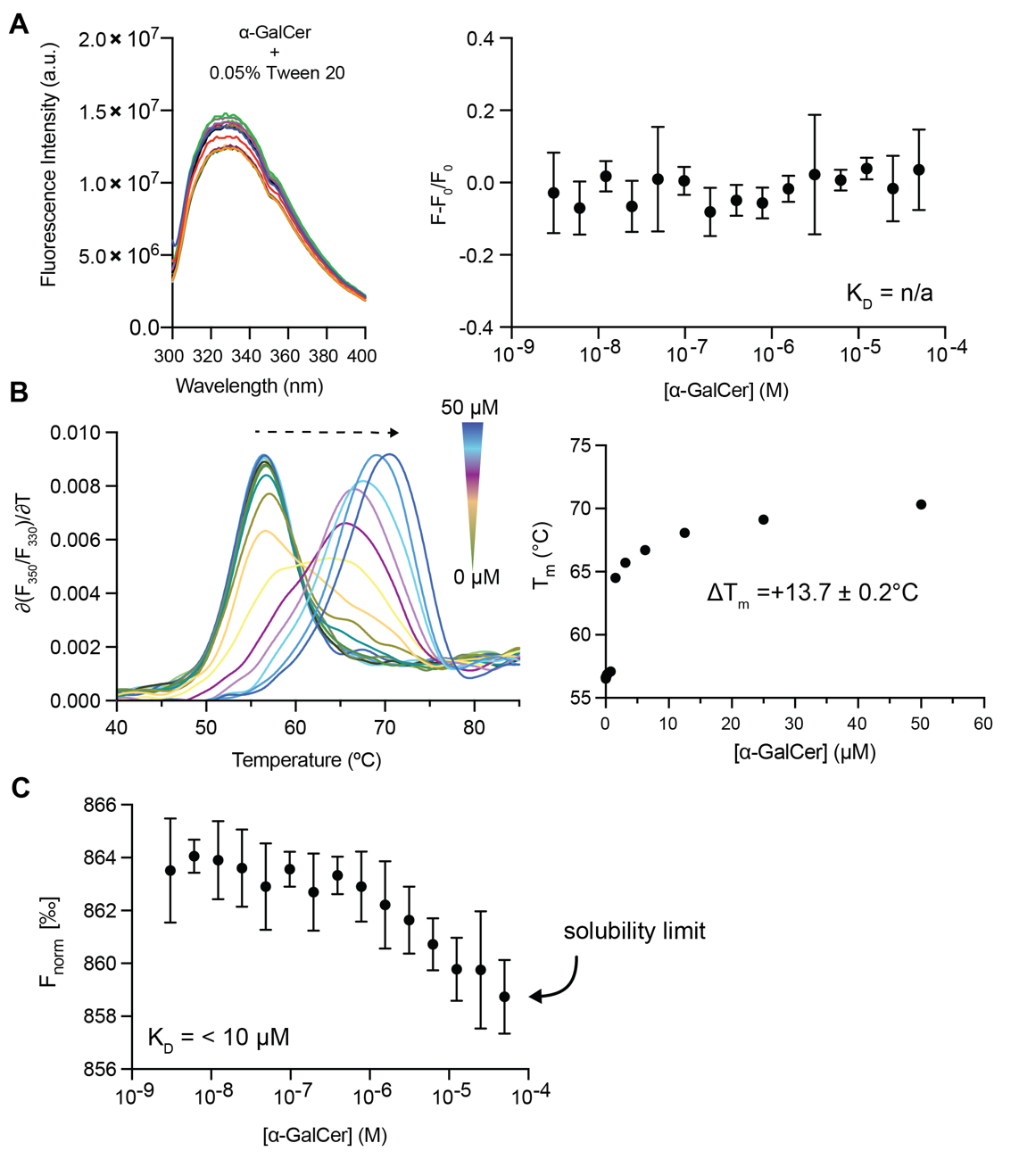


**Supplementary Figure S11. Binding of α-GalCer to hCD1d by ITF, MST, and nanoDSF in the presence of Tween 20.** (A) ITF data showing the change in Trp fluorescence (F-F_0_/F_0_ where F_0_ is initial fluorescence in the absence of detergent) as a function of increasing concentrations of α-GalCer in the presence of 100 nM hCD1d with 0.05% Tween 20 (black circles) at 25°C. Fits of the binding isotherms are as shown with a red line. Each data is mean ± standard deviation for three replicates. (B) NanoDSF data showing the change in the first derivative of the Trp fluorescence ratio, ∂(F_350_/F_330_)/ ∂T, as a function of increasing concentrations of α-GalCer with 1 µM hCD1d with 0.05% Tween 20. The color gradient denotes the concentration range of α-GalCer where the DSF spectra are color coded. The dotted arrows highlight an α-GalCer-dependent increase in the melting temperature (T_m_) of hCD1d. ΔT_m_ is defined as the T_m_ difference between CD1d in the absence and presence of 50 µM α-GalCer. Each data is mean ± standard deviation for three replicates. (C) MST data showing the change in normalized fluorescence signal (F_norm_, black circles) as a function of increasing concentrations of α-GalCer with 100 nM AF647-labeled hCD1d with 0.05% Tween 20 at 25°C. Each data is mean ± standard deviation for three replicates.

**
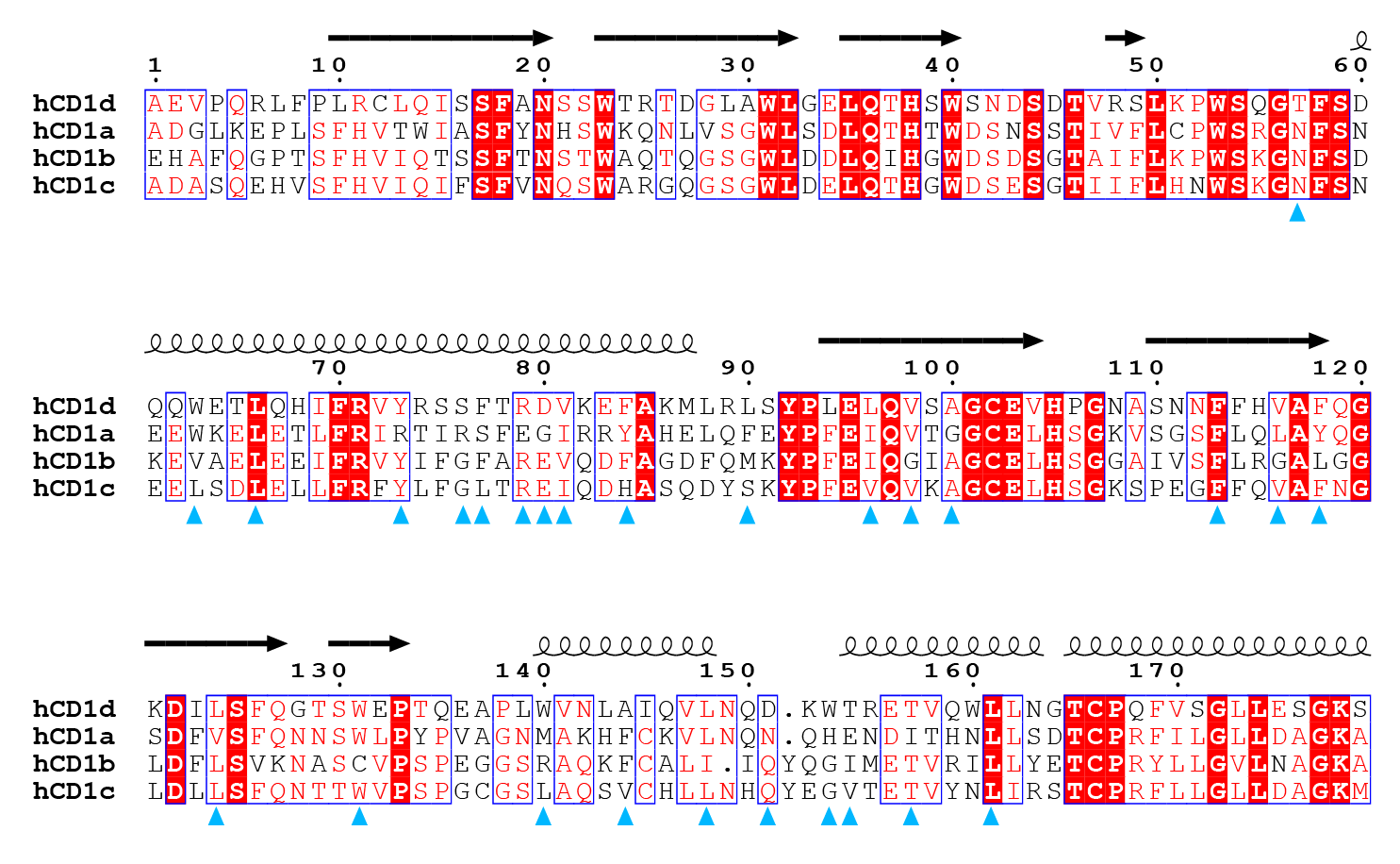
**

**Supplementary Figure S12. Comparison of predicted detergent binding residues across human CD1 isoforms.** Protein sequence alignment for the ectodomain of human CD1 molecules with Clustal Omega v1.2.4 and visualized in ESPript v3.0. Sequences used: hCD1a (UniProt #P06126), hCD1b (UniProt #P29016), hCD1c (UniProt #P29017), and hCD1d (UniProt #P15813). The blue triangles represent hCD1d residues predicted to interact with detergents based on Chai-1 models (see Table S2).

**Table S1. Chai-1 model confidence metrics for the detergent/CD1d ortholog complex models**

| **Detergent** | **human  CD1d** | **mouse CD1d1** | **rat CD1d** | **rhesus CD1d** | **pig CD1d** | **bovine CD1d** |
| --- | --- | --- | --- | --- | --- | --- |
| Tween 20 | agg = 0.85 ipTM = 0.84 pTM = 0.89 | agg = 0.84 ipTM = 0.83 pTM = 0.87 | agg = 0.83 ipTM = 0.82  pTM = 0.86 | agg = 0.84 ipTM = 0.83  pTM = 0.87 | agg = 0.84 ipTM = 0.83  pTM = 0.87 | agg = 0.83 ipTM = 0.82 pTM = 0.87 |
| Tween 80 | agg = 0.84 ipTM = 0.83 pTM = 0.88 | agg = 0.83 ipTM = 0.82 pTM = 0.87 | agg = 0.81 ipTM = 0.80 pTM = 0.85 | agg = 0.82 ipTM = 0.81 pTM = 0.86 | agg = 0.83 ipTM = 0.82 pTM = 0.87 | agg = 0.82 ipTM = 0.81 pTM = 0.86 |
| ODG | agg = 0.91 ipTM = 0.91 pTM = 0.93 | agg = 0.89  ipTM = 0.88 pTM = 0.92 | agg = 0.88 ipTM = 0.87 pTM = 0.91 | agg = 0.89 ipTM = 0.89 pTM = 0.92 | agg = 0.89 ipTM = 0.88 pTM = 0.91 | agg = 0.89 ipTM = 0.89 pTM = 0.92 |
| Triton X-100 | agg = 0.90 ipTM = 0.90 pTM = 0.93 | agg = 0.89 ipTM = 0.88 pTM = 0.91 | agg = 0.87 ipTM = 0.86 pTM = 0.90 | agg = 0.89 ipTM = 0.88 pTM = 0.91 | agg = 0.88 ipTM = 0.88 pTM = 0.91 | agg = 0.89 ipTM = 0.88 pTM = 0.91 |
| DDM | agg = 0.91 ipTM = 0.91 pTM = 0.93 | agg = 0.92 ipTM = 0.92 pTM = 0.94 | agg = 0.90 ipTM = 0.89 pTM = 0.93 | agg = 0.91 ipTM = 0.91 pTM = 0.93 | agg = 0.9 ipTM = 0.90 pTM = 0.93 | agg = 0.93 ipTM = 0.92 pTM = 0.95 |
| Tyloxapol | agg = 0.77 ipTM = 0.76 pTM = 0.80 | agg = 0.81 ipTM = 0.80 pTM = 0.84 | agg = 0.81 ipTM = 0.81 pTM = 0.85 | agg = 0.77 ipTM = 0.76  pTM = 0.81 | agg = 0.79 ipTM = 0.78 pTM = 0.83 | agg = 0.78  ipTM = 0.77 pTM = 0.82 |
| NP-40 | agg = 0.92 ipTM = 0.92 pTM = 0.94 | agg = 0.89 ipTM = 0.89 pTM = 0.92 | agg = 0.88 ipTM = 0.88  pTM = 0.91 | agg = 0.92 ipTM = 0.91 pTM = 0.93 | agg = 0.91 ipTM = 0.90 pTM = 0.93 | agg = 0.91 ipTM = 0.90 pTM = 0.93 |
| Brij-35 | agg = 0.85 ipTM = 0.84 pTM = 0.88 | agg = 0.83 ipTM = 0.82 pTM = 0.87 | agg = 0.83 ipTM = 0.82 pTM = 0.87 | agg = 0.83 ipTM = 0.82 pTM = 0.87 | agg = 0.82 ipTM = 0.81 pTM = 0.86 | agg = 0.83  ipTM = 0.82 pTM = 0.87 |
| CTAB | agg = 0.92 ipTM = 0.94 pTM = 0.91 | agg = 0.91 ipTM = 0.90 pTM = 0.93 | agg = 0.90 ipTM = 0.89 pTM = 0.92 | agg = 0.92 ipTM = 0.92 pTM = 0.94 | agg = 0.90  ipTM = 0.90 pTM = 0.92 | agg = 0.91 ipTM = 0.91 pTM = 0.93 |
| Deoxycholate | agg = 0.89 ipTM = 0.88 pTM = 0.92 | agg = 0.87 ipTM = 0.87 pTM = 0.91 | agg = 0.88 ipTM = 0.87  pTM = 0.91 | agg = 0.87 ipTM = 0.86 pTM = 0.90 | agg = 0.88 ipTM = 0.87 pTM = 0.90 | agg = 0.88 ipTM = 0.87 pTM = 0.91 |
| SDS | agg = 0.92 ipTM = 0.91 pTM = 0.94 | agg = 0.90 ipTM = 0.90 pTM = 0.92 | agg = 0.90 ipTM = 0.89 pTM = 0.92 | agg = 0.91 ipTM = 0.91 pTM = 0.94 | agg = 0.90 ipTM = 0.89 pTM = 0.92 | agg = 0.91 ipTM = 0.91 pTM = 0.94 |
| LDAO | agg = 0.92 ipTM = 0.92 pTM = 0.94 | agg = 0.91 ipTM = 0.90 pTM = 0.93 | agg = 0.90 ipTM = 0.90 pTM = 0.93 | agg = 0.92 ipTM = 0.92 pTM = 0.94 | agg = 0.90 ipTM = 0.90 pTM = 0.92 | agg = 0.92 ipTM = 0.92 pTM = 0.94 |
| CHAPS | agg = 0.84 ipTM = 0.83 pTM = 0.87 | agg = 0.85 ipTM = 0.84  pTM = 0.88 | agg = 0.86  ipTM = 0.85 pTM = 0.89 | agg = 0.85 ipTM = 0.84 pTM = 0.88 | agg = 0.85 ipTM = 0.84  pTM = 0.89 | agg = 0.84 ipTM = 0.83  pTM = 0.88 |

agg = Chai-1 aggregate score

**SMILES strings used for Chai-1 modeling:**

Tween 20

OCCOCCOCCOCCOCCOC(COCCOCCOCCOCCOCCOC(CCCCCCCCCCC)=O)C[C@@H]1[C@@H](OCCOCCOCCOCCOCCO)[C@H](OCCOCCOCCOCCOCCO)CO1

Tween 80

OCCOCCOCCOCCOCCOC(COCCOCCOCCOCCOCCOC(CCCCCCC/C=C\CCCCCCCC)=O)C[C@@H]1[C@@H](OCCOCCOCCOCCOCCO)[C@H](OCCOCCOCCOCCOCCO)CO1

ODG

O[C@H]1[C@H](O)[C@@H](O)[C@H](OCCCCCCCC)O[C@@H]1CO

Triton X-100

CC(C(C)(C)C)(C)C1=CC=C(OCCOCCOCCOCCOCCOCCOCCOCCOCCOCCO)C=C1

DDM

CCCCCCCCCCCCO[C@H]1[C@@H]([C@H]([C@@H]([C@H](O1)CO)O[C@@H]2[C@@H]([C@H]([C@@H]([C@H](O2)CO)O)O)O)O)O

Tyloxapol

OCCOCCOCCOCCOCCOCCOCCOCCCCCOCCOC1=C(C2=C(OCCOCCOCCOCCOCCOCCOCCOCCOCCOCCO)C(C3=CC(C(C)(C)C(C(C)(C)C)(C)C)=CC=C3OCCOCCOCCOCCOCCOCCOCCOCCOCCOCCO)=CC(C(C)(C)C(C)(C)C(C)(C)C)=C2)C=C(C(C)(C)C(C)(C)C(C)(C)C)C=C1C4=CC(C(C(C)(C)C(C)(C)C)(C)C)=CC(C5=C(OCCOCCOCCOCCOCCOCCOCCOCCOCCOCCO)C=CC(C(C)(C)C(C)(C)C(C)(C)C)=C5)=C4OCCOCCOCCOCCOCCOCCOCCOCCOCCOCCO

NP-40

CCCCCCCCCC1=CC=C(OCCOCCOCCOCCOCCOCCOCCOCCOCCO)C=C1

Brij-35

CCCCCCCCCCCCOCCOCCOCCOCCOCCOCCOCCOCCOCCOCCOCCOCCOCCOCCOCCOCCOCCOCCOCCOCCOCCOCCOCCO

CTAB

CCCCCCCCCCCCCCCC[N+](C)(C)C

Deoxycholate

C[C@H](CCC(=O)[O-])[C@H]1CC[C@@H]2[C@@]1([C@H](C[C@H]3[C@H]2CC[C@H]4[C@@]3(CC[C@H](C4)O)C)O)C

SDS

CCCCCCCCCCCCOS(=O)([O-])=O

LDAO

CCCCCCCCCCCC[N+](C)(C)[O-]

CHAPS

C[C@H](CCC(=O)NCCC[N+](C)(C)CCCS(=O)(=O)[O-])[C@H]1CC[C@@H]2[C@@]1([C@H](C[C@H]3[C@H]2[C@@H](C[C@H]4[C@@]3(CC[C@H](C4)O)C)O)O)C

α-Galactosylceramide

CCCCCCCCCCCCCCCCCCCCCCCCCC(=O)N[C@@H](CO[C@@H]1[C@@H]([C@H]([C@H]([C@H](O1)CO)O)O)O)[C@@H]([C@@H](CCCCCCCCCCCCCC)O)O

18:1 lysophosphatidylcholine

CCCCCCCC/C=C/CCCCCCCC(=O)OC[C@H](COP(=O)(O)OCC[N+](C)(C)C)O

**Protein Sequences used for Chai-1 modeling:**

human β2M (UniProt #P61769):

IQRTPKIQVYSRHPAENGKSNFLNCYVSGFHPSDIEVDLLKNGERIEKVEHSDLSFSKDWSFYLLYYTEFTPTEKDEYACRVNHVTLSQPKIVKWDRDM

human CD1d (UniProt #P15813):

AEVPQRLFPLRCLQISSFANSSWTRTDGLAWLGELQTHSWSNDSDTVRSLKPWSQGTFSDQQWETLQHIFRVYRSSFTRDVKEFAKMLRLSYPLELQVSAGCEVHPGNASNNFFHVAFQGKDILSFQGTSWEPTQEAPLWVNLAIQVLNQDKWTRETVQWLLNGTCPQFVSGLLESGKSELKKQVKPKAWLSRGPSPGPGRLLLVCHVSGFYPKPVWVKWMRGEQEQQGTQPGDILPNADETWYLRATLDVVAGEAAGLSCRVKHSSLEGQDIVLYW

Mouse β2M (UniProt #P01887):

IQKTPQIQVYSRHPPENGKPNILNCYVTQFHPPHIEIQMLKNGKKIPKVEMSDMSFSKDWSFYILAHTEFTPTETDTYACRVKHASMAEPKTVYWDRDM

Mouse CD1d1 (UniProt #P11609):

SEAQQKNYTFRCLQMSSFANRSWSRTDSVVWLGDLQTHRWSNDSATISFTKPWSQGKLSNQQWEKLQHMFQVYRVSFTRDIQELVKMMSPKEDYPIEIQLSAGCEMYPGNASESFLHVAFQGKYVVRFWGTSWQTVPGAPSWLDLPIKVLNADQGTSATVQMLLNDTCPLFVRGLLEAGKSDLEKQEKPVAWLSSVPSSADGHRQLVCHVSGFYPKPVWVMWMRGDQEQQGTHRGDFLPNADETWYLQATLDVEAGEEAGLACRVKHSSLGGQDIILYW

Rat β2M (UniProt #P07151):

IQKTPQIQVYSRHPPENGKPNFLNCYVSQFHPPQIEIELLKNGKKIPNIEMSDLSFSKDWSFYILAHTEFTPTETDVYACRVKHVTLKEPKTVTWDRDM

Rat CD1d (UniProt #Q63493):

QSEVQQNYTFGCLQISSFANRSWSRTDSVVWLGDLQTHRWSNDSDTISFTKPWSQGKFSNQQWEKLQHMFQVYRTSFTRDIKEIVKMMSPKEDYPIEVQLSAGCEMYPGNASESFLHVAFQGEYVVRFHGTSWQKVPEAPSWLDLPIKMLNADEGTRETVQILLNDTCPQFVRGLLEAGKPDLEKQEKPVAWLSRGPNPAHGHLQLVCHVSGFHPKPVWVMWMRGDQEQGGTHRGDILPNADETWYLQATLDVEAGDEAGLACRVKHSSLEGQDIILYW

Rhesus β2M (UniProt #Q6V7J5):

IQRTPKIQVYSRHPPENGKPNFLNCYVSGFHPSDIEVDLLKNGEKMGKVEHSDLSFSKDWSFYLLYYTEFTPNEKDEYACRVNHVTLSGPRTVKWDRDM

Rhesus CD1d (UniProt #F6TR81):

AEVPQRLFPLRCLQISSFANSNWTRTDGLAWLGELQTHSWSNDSDTIRSLKPWSQGTFSDQQWEALQRVFRVYRSSFTRDVKEFAKMLRLAYPMELQVSAGCEVHPGNASHNFFHVAFQGSDILSFQGTSWEPAQEAPLWVNLAIQVLNQDNWTKETVQWLLNDTCPQFVSGLLESGKSELEKQVKPKAWLSRGPSPGPGRLQLVCHVSGFYPKPVWVKWMRGEQEQQGTQRGDILPNADETWYLRATLEVAAGEAAGLSCRVKHSSLEGQDIILYW

Pig β2M (UniProt #Q07717):

VARPPKVQVYSRHPAENGKPNYLNCYVSGFHPPQIEIDLLKNGEKMNAEQSDLSFSKDWSFYLLVHTEFTPNAVDQYSCRVKHVTLDKPKIVKWDRDH

Pig CD1d (UniProt #A0ZQ05):

LPASPRFFPLRCLQISSFANSSWTRTDGLGWVGELQAYTWRNDSDTIVFLKPWSQGTLSDQLANQLQHIFKGYRSSFTRDIREFVKMLGSDYPFEIQISAGCEVLSGNSSESFLHSAFQGTDIMSFQGTSWVSAPDAPPWMQRVCRVLNEDQGTKETVQWLLYDICPQFVRGVLETGKSELEKQVKPEAWLSSGPTPGPGRLLLVCHVSGFYPKPVWVMWMRGEQEQPGTQQGDILPHADGTWYLRVTLDVAAGEASGLSCRVKHSSLGDQDIILYW

Bovine β2M (UniProt #P01888):

IQRPPKIQVYSRHPPEDGKPNYLNCYVYGFHPPQIEIDLLKNGEKIKSEQSDLSFSKDWSFYLLSHAEFTPNSKDQYSCRVKHVTLEQPRIVKWDRDL

Bovine CD1d (UniProt #A1L565):

SPAPQTPFSFQGLQISSFANRSWTRTDGLAWLGELQPYTWRNESDTIRFLKPWSRGTFSDQQWEQLQHTLLVYRSSFTRDIWEFVEKLHVEYPLEIQIATGCELLPRNISESFLRAAFQGRDVLSFQGMSWVSAPDAPPFIQEVIKVLNQNQGTKETVHWLLHDIWPELVRGVLQTGKSELEKQVKPEAWLSSGPSPGPGRLLLVCHVSGFYPKPVRVMWMRGEQEEPGTRQGDVMPNADSTWYLRVTLDVAAGEVAGLSCQVKHSSLGDQDIILYW

**Table S2. Conserved interaction residues in Chai-1 models of detergent/hCD1d complexes relative to α-GalCer/hCD1d**

| **hCD1d Residue** | **Type of interaction** | **# of complexes (out of 13)** | **Interacts with α-GalCer?** | **Detergent** |
| --- | --- | --- | --- | --- |
| L148 | Hydrophobic | 10/13 | ❌ | Brij35, CHAPS, CTAB, DDM, LDAO, NP40, ODG, SDS, Tween20, Tyloxapol |
| F77 | Hydrophobic | 10/13 | ❌ | Brij35, CHAPS, DDM, Deoxycholate, NP40, ODG, SDS, TritonX100, Tween80, Tyloxapol |
| L96 | Hydrophobic | 9/13 | ❌ | Brij35, CTAB, LDAO, NP40, ODG, SDS, TritonX100, Tween80, Tyloxapol |
| D80 | Hydrophobic | 8/13 | ❌ | Brij35, CHAPS, CTAB, DDM, Deoxycholate, ODG, TritonX100, Tyloxapol |
| D80 | Hydrogen Bond | 3/13 | ❌ | Brij35, DDM, ODG |
| D80 | Salt Bridge | 1/13 | ❌ | CTAB |
| S76 | Hydrophobic | 8/13 | ✓ | CHAPS, DDM, Deoxycholate, LDAO, ODG, Tween20, Tween80, Tyloxapol |
| S76 | Hydrogen Bond | 8/13 | ✓ | CHAPS, DDM, Deoxycholate, LDAO, ODG, Tween20, Tween80, Tyloxapol |
| L90 | Hydrophobic | 7/13 | ✓ | Brij35, CTAB, DDM, NP40, SDS, Tween20, Tween80 |
| W140 | Hydrophobic | 7/13 | ❌ | Brij35, DDM, SDS, TritonX100, Tween20, Tween80, Tyloxapol |
| F84 | Hydrophobic | 7/13 | ✓ | CHAPS, CTAB, DDM, TritonX100, Tween20, Tween80, Tyloxapol |
| W131 | Hydrophobic | 7/13 | ❌ | CHAPS, CTAB, DDM, NP40, SDS, Tween20, Tween80 |
| Y73 | Hydrophobic | 5/13 | ✓ | CHAPS, Deoxycholate, NP40, Tween20, Tyloxapol |
| Y73 | Hydrogen Bond | 3/13 | ✓ | CHAPS, NP40, Tween20 |
| V81 | Hydrophobic | 4/13 | ❌ | Brij35, TritonX100, Tween20, Tyloxapol |
| L124 | Hydrophobic | 4/13 | ✓ | Brij35, NP40, Tween80, Tyloxapol |
| T154 | Hydrophobic | 4/13 | ✓ | CHAPS, CTAB, NP40, ODG |
| T154 | Hydrogen Bond | 2/13 | ✓ | CHAPS, ODG |
| R79 | Hydrophobic | 4/13 | ❌ | DDM, ODG, Tween80, Tyloxapol |
| R79 | Hydrogen Bond | 4/13 | ❌ | DDM, ODG, Tween80, Tyloxapol |
| W153 | Hydrophobic | 4/13 | ❌ | CHAPS, CTAB, Deoxycholate, Tyloxapol |
| V116 | Hydrophobic | 3/13 | ✓ | CHAPS, TritonX100, Tween80 |
| A144 | Hydrophobic | 2/13 | ❌ | TritonX100, Tyloxapol |
| L161 | Hydrophobic | 2/13 | ✓ | Tween20, Tween80 |
| F118 | Hydrophobic | 2/13 | ✓ | NP40, Tween80 |
| D151 | Hydrophobic | 2/13 | ❌ | CHAPS, CTAB |
| D151 | Hydrogen Bond | 1/13 | ❌ | CHAPS |
| D151 | Salt Bridge | 2/13 | ❌ | CHAPS, CTAB |
| T157 | Hydrophobic | 1/13 | ✓ | Brij35 |
| T157 | Hydrogen Bond | 1/13 | ✓ | Brij35 |
| W63 | Hydrophobic | 1/13 | ❌ | Tyloxapol |
| F114 | Hydrophobic | 1/13 | ❌ | Tyloxapol |
| V98 | Hydrophobic | 1/13 | ❌ | Tyloxapol |
| A100 | Hydrophobic | 1/13 | ❌ | Tyloxapol |
| L66 | Hydrophobic | 1/13 | ✓ | Tyloxapol |
| T57 | Hydrophobic | 1/13 | ❌ | Tyloxapol |
| T57 | H-Bond | 1/13 | ❌ | Tyloxapol |
| V118 | Hydrophobic | 1/13 | ❌ | Tyloxapol |
| F70 | Hydrophobic | 1/13 | ✓ | Tyloxapol |
| V47 | Hydrophobic | 1/13 | ✓ | Tyloxapol |
| F169 | Hydrophobic | 1/13 | ✓ | Tyloxapol |
| I69 | Hydrophobic | 1/13 | ✓ | Tween80 |
| L94 | Hydrophobic | 1/13 | ❌ | Tween80 |
| L29 | Hydrophobic | 1/13 | ❌ | Tween20 |
| L29 | H-Bond | 1/13 | ❌ | Tween20 |
| V72 | Hydrophobic | 1/13 | ❌ | Deoxycholate |
